# Assessment of Food By-Products’ Potential for Simultaneous Binding of Aflatoxin B1 and Zearalenone

**DOI:** 10.3390/toxins13010002

**Published:** 2020-12-22

**Authors:** Laurentiu Mihai Palade, Madalina Ioana Dore, Daniela Eliza Marin, Mircea Catalin Rotar, Ionelia Taranu

**Affiliations:** National Research Development Institute for Animal Biology and Nutrition, 077015 IBNA Balotesti, Romania; dore.madalina@ibna.ro (M.I.D.); daniela.marin@ibna.ro (D.E.M.); catalin.rotar@ibna.ro (M.C.R.); ionelia.taranu@ibna.ro (I.T.)

**Keywords:** mycotoxins, food by-products, grape seed meal, seabuckthorn meal, waste recycling, decontamination, adsorption

## Abstract

In this study, eight food by-products were investigated as biosorbent approaches in removing mycotoxin load towards potential dietary inclusion in animal feed. Among these food-derived by-products, grape seed (GSM) and seabuckthorn (SBM) meals showed the most promising binding capacity for Aflatoxin B1 (AFB1) and Zearalenone (ZEA), measured as percent of adsorbed mycotoxin. Furthermore, we explored the mycotoxin sequestering potential by screening the effect of time, concentration, temperature and pH. Comparative binding efficacy was addressed by carrying out adsorption experiments in vitro. The highest mycotoxin adsorption was attained using 30 mg of by-product for both GSM (85.9% AFB1 and 83.7% ZEA) and SBM (68% AFB1 and 84.5% ZEA). Optimal settings for the experimental factors were predicted employing the response surface design. GSM was estimated to adsorb AFB1 optimally at a concentration of 29 mg/mL, pH 5.95 and 33.6 °C, and ZEA using 28 mg/mL at pH 5.76 and 31.7 °C. Favorable adsorption of AFB1 was estimated at 37.5 mg of SBM (pH 8.1; 35.6 °C), and of ZEA at 30.2 mg of SBM (pH 5.6; 29.3 °C). Overall, GSM revealed a higher binding capacity compared with SBM. In addition, the two by-products showed different specificity for the binary–mycotoxin system, with SBM having higher affinity towards ZEA than AFB1 (K_f_ = 0.418 and 1/n = 0.213 vs. K_f_ = 0.217 and 1/n = 0.341) and GSM for AFB1 in comparison with ZEA (K_f_ = 0.367 and 1/n = 0.248 vs. K_f_ = 0.343 and 1/n = 0.264). In conclusion, this study suggests that GSM and SBM represent viable alternatives to commercial biosorbent products.

## 1. Introduction

The agricultural industry and food processing industry generate waste in multiple forms, under various conditions and through a plethora of processes. Containing mainly biodegradable organic matter, the accumulation of waste materials exerts increasing detrimental effects on the environment [1,2,3]. Various estimates increasingly suggest that agricultural waste management translates in high removal costs [1]. One way of addressing this impeding issue has been identified as “recycling”. As such, several processes have been explored worldwide in order to effectively reuse various by-products [4,5]. In this context, food waste could be regarded as alternative sources used in animal nutrition, diminishing food competition between humans and animals [5]. Recent scientific advancement has been focused on characterizing various agricultural by-products for further utilization as animal feed, including potential effects on health and animal performance [6]. In this context, various food by-products have been included in the Catalogue of feed materials (Commission Regulation No 68/2013 of 16 January 2013), further updated in 2017 (Commission Regulation (EU) 2017/1017) [7,8]. Moreover, the recycling of waste is one of the European Commission’s priority of the Horizon 2020 European research program 12: climate action, environment, resource efficiency and raw materials-WASTE-7-2015: Ensuring sustainable use of agricultural waste, co-products, and by-products (2008/98/EC directive). Specifically, EU encourages a sustainable and continuous recycling of grape marc to avoid accumulation, its utilization as fertilizers being unfavorable due to the anti-germination activity of polyphenols.

Many plant by-products rich in bioactive compounds have been exploited for nutritional purposes for both humans and animals [9,10,11]. For example, several dietary polyphenol-rich by-products, such as grape pomace, were found to effectively improve the physiology and biochemistry of the gut in broiler chicks and piglets, exerting better nutrient absorption, as well as increased protection against diseases [12,13,14]. Similarly, as a source of ω-3 polyunsaturated fatty acids (PUFAs), seabuckthorn by-products have shown to possess health promoting properties [15]. Dannenberger et al. (2018) investigated the sea buckthorn pomace supplementation in the diet of growing German Landrace pigs and observed moderate effects on fatty acid metabolism [16]. Sharma et al. (2018) found that a 0.5% level of seabuckthorn leaf meal supplementation of turkey diet may improve their production performance [17].

Besides the potential beneficial effects of including agricultural by-products in animal diets, the focus has recently turned towards their application in mycotoxin removal or mycotoxin mitigating effects [18]. Broadly studied, mycotoxin load and co-contamination in food and feed represent a major threat to a wide range of animal species, especially to humans [19,20]. Widely described in literature, aflatoxins and zearaleone are common mycotoxins that occur naturally being fungal secondary metabolites produced by *Aspergilus* sp. and *Fusarium* sp., which contaminate animal and human feed and food. They represent a major problem within the frame of world economic losses, but more importantly a pressing health concern due to their toxic impact on animals and humans [19,21,22]. Thus, aflatoxin B1 (AFB1) presents the highest toxic potential being classified by the International Agency for Research on Cancer as one of the primary carcinogenic compounds in the development of liver cancer, while zearalenone’s toxicity affects the reproductive system in both animals and humans [23,24]. Different approaches have been elaborated in order to address the task of minimizing the impact of mycotoxin co-occurrence in food and feed and to alleviate their harmful effects [25,26]. One of the most applied methods is the inclusion of different adsorbents into animal feed, which bind the mycotoxins before feed intake, but mainly during digestion [21]. Various compounds have been explored as mycotoxin adsorbents including clays, activated carbons, synthetic polymers and indigestible carbohydrates [27]. Nonetheless, owing to the complex chemical structures and diverse properties of mycotoxins, the application of many mycotoxin adsorbents regulated by the Commission Regulation, No 386/2009 [28] render inconclusive results. This stems from their limited specificity, resulting in the incapacity of binding mycotoxins that are structurally different [12,27].

There are several studies reporting that carbohydrates manifested toxin binder properties. For example, Banlunara et al. (2005) described glucomannan, a polysaccharide from yeast cell walls of *Saccharomyces cervisiae,* consisting in functional carbohydrates which contains a large surface and number of pores of different sizes to trap a wide range of chemicals as an excellent AFB1 binder [29]. Also, Solis-Cruz et al. (2019) showed that cellulose has a great potential to adsorb AFB1 by electrostatic attractions and hydrogen bonding resulting in formation of a mycotoxin monolayer on its surface [30]. Waste by-products contain also other bioactive compounds such as polyphenols, polyunsaturated fatty acids (PUFA), etc. with binding or mitigating potential. Thus, Lu et al. (2017) demonstrated that polyphenols from fermented tea form with AFB1 a C-AFB1 complex and consequently inhibited the AFB1 absorption, facilitating its faeces elimination and the reduction of liver injury [24]. A recent study of Avantaggiato et al. (2014) also reported the in vitro sequestering of AFB1 and ZEA by grape marc through adsorption tests in liquid media [12]. Accordingly, the addition of agricultural wastes as mycotoxin adsorbents to contaminated feed is regarded as safe approach to act against the toxic effects of mycotoxins to livestock [12,27]. In recent studies, there has been substantial evidence that the binding capacity of plant-derived compounds is strongly related to surface interaction with the mycotoxin [31], with high involvement of the structural characteristics of the interacting molecules [24]. In line with this, previous reports highlighted the importance of adsorbent particle size, medium pH, and temperature associated with the adsorption process [12,22,32,33].

The aim of the present study was to investigate the biosorbent capacity of several food by-products and their suitability towards reducing mycotoxin contamination. The choice of the by-products was done for both scientific and practical reasons. There is a growing interest in investigating the potential of by-products as mycotoxin binders, and there are significant quantities of agricultural products and by-products that are produced annually in many agricultural countries.

The secondary objective of our study was to determine the influence of the in vitro tested conditions (incubation time, food by-product concentration, medium pH, and incubation temperature) on the binary-mycotoxin binding process in liquid media. There are several studies reporting the mycotoxin adsorption capacity of grape pomace (pulp and skin) [12,27]. However, herein we evaluated the mycotoxin adsorption potential of a less investigated by-product, e.g grape seed meal, which is obtained after oil extraction. Moreover, to our knowledge, seabuckthorn meal has not been investigated so far with regard to its bio-adsorbent capacity. These by-products were selected as bearing the highest binding capacity among the tested ones, and were further subjected to process optimisation, which was carried out to simultaneously assess the effect of the tested parameters on mycotoxin removal. The estimation of favorable conditions related to the adsorption process could be regarded as a novelty and an obvious step towards industrial application.

## 2. Results

### 2.1. UHPLC Results

AFB1 and ZEA residues have been reported previously [12,27]. Mycotoxin analysis is continually improved to meet the increasing requirements for reliable and accurate methods [34]. The use of UHPLC brings a series of advantages over the classical HPLC methods, such as increased resolution, shorter analysis time, and mainly reduced solvent use [35]. 

In this study, we developed fast and accurate UHPLC method, which allowed the simultaneous determination of the two mycotoxins.

**Figure 1:**
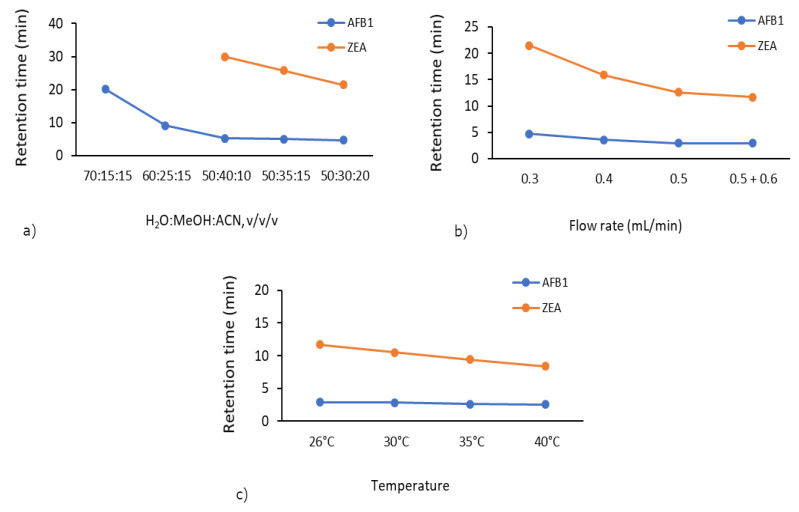
Method optimisation—variation of total analysis time according to (**a**) mobile phase ratio, (**b**) flow rate, and (**c**) column temperature.

The importance of mobile phase composition originates from its effect on the separation of target compounds and the corresponding peak response [36]. In order to select the most appropriate mobile phase ratio, a range of mobile phase ratios varying from 70:15:15/60:25:15/50:40:10/50:35:15/50:30:20 were tested (Supplementary Figures S1–S5). The suitable ratio of 50:30:20 (*v*/*v*/*v*) for the H_2_O:MeOH:ACN mobile phase was selected for the determination of AFB1 and ZEA residues at a flow rate of 0.3 mL/min. As shown in Figure 1a, we observed a decrease in the total analysis time. Initially, we did not detect ZEA due to the 30 min analysis time. However, using the 50:30:20 mobile phase ratio, the elution of ZEA was improved (21.5 min). 

By varying the flow rate from 0.3 mL/min to 0.5 mL/min combined with a ramp to 0.6 mL/min, the retention time of ZEA decreased from 21.5 to 11.7 min (Figure 1b). Accordingly, the optimum flow rate of 0.5 + 0.6 mL/min was selected at a mobile phase ratio of 50:30:20 due to obvious improved retention time and chromatogram shape as compared to the range 0.3–0.5 mL/min (Supplementary Figures S5–S8). 

Subsequently, by increasing the column temperature from 26 to 40 °C, the retention time of ZEA decreased from 11.7 min to just 8.4 min (Figure 1c). A suitable temperature (40 °C) was selected to carry out the mycotoxin adsorption experiments, which retained good peak area and peak shape (Supplementary Figures S8–S11).

The final chromatographic separation of the two mycotoxins was considered to have achieved good resolution in 12 min (total analysis time), at 40 °C column temperature on an Acclaim™ 120 C18 column, using H_2_O:MeOH:ACN in the ratio of 50:30:20 (*v*/*v*/*v*) as mobile phase, and a flow rate gradient of 0.5 mL/min (0–5 min), 0.6 mL/min (5.01–10 min), and finally 0.5 mL/min (10.01–12 min).

### 2.2. Method Validation Results

**Figure 2:**
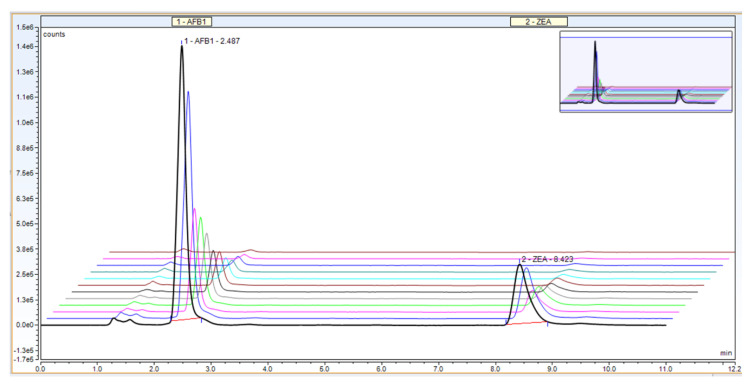
Example chromatogram for UHPLC calibration curve—mobile ratio of 50:30:20, flow rate 0.5 + 0.6 mL/min, column temperature 40 °C.

Selectivity of the method, a very important parameter in validation, is the ability to separate different compounds (including impurities) present in the sample. Our proposed UHPLC method allows for the quantitative determination of AFB1 and ZEA simultaneously, revealing a proper separation of the mycotoxins and no interference between the two compounds (AFB1 at 2.4 min and ZEA at 8.4 min, respectively; Figure 2). 

Generally, LOD is the lowest concentration of a compound that produces a detector signal easily distinguished from the baseline and is calculated as three times the baseline noise, whereas LOQ is the lowest concentration of a compound that can be accurately quantified and is calculated as ten times the baseline noise. In our study, LOD (S/N = 3) and LOQ (S/N = 10) were 0.15 μg/mL and 0.50 μg/mL for both AFB1 and ZEA, respectively.

Method linearity was evaluated based on the calibration curves and showed very good coefficients of determination (R^2^) within the selected concentration range (0.05–6.25 µg/mL) for both AFB1 and ZEA (>0.998). Method accuracy, the difference between the reference true value and the measured value, was determined on spiked blank samples at 5 μg/mL mycotoxin concentration. The precision of the method includes repeatability (intra-day assay) and reproducibility (inter-day assay). The method revealed intermediate precision for AFB1 and ZEA, with repeatability that ranged from 1.58 to 1.61% and reproducibility from 2.41 and 2.83%, respectively. These values are in agreement with EU guideline 96/23/EC and FDA guideline for a validated analytical method [37,38]. 

Our results are presented in Table 1 and showed that the UHPLC method was validated and reliable for the quantification of AFB1 and ZEA residues in liquid media.

### 2.3. Effect of Influencing Conditions on the Binding Proces

#### 2.3.1. Effect of Incubation Time 

**Figure 3:**
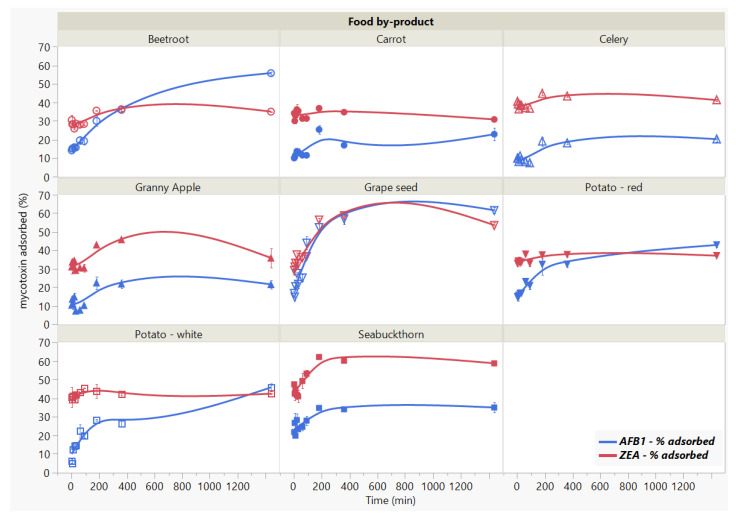
Effect of incubation time on AFB1 and ZEA binding rates. Binding experiments were performed at constant pH (7) and temperature (37 °C), using 5 mg/mL residue concentration and 5 μg/mL mycotoxin concentration.

We studied the influence of incubation time on mycotoxins binding by the 8 food by-products for a period of 24 h (Figure 3). The significant interaction effect (Food by-product × Time, *p* < 0.0001 *) indicates that the reduction of adsorption for both mycotoxins in time (Time, *p* < 0.0001 *) is reached differently by the analysed by-products (Food by-product, *p* < 0.0001 *). The AFB1 adsorption kinetics reflects rapid uptake (<5 min) by the food by-products, accompanied by slight changes in the initial stages of the process until 60 min. We observed a further increase in the AFB1 adsorption rate after the 90 min threshold, rendering noticeable differences at the final stage of the studied contact time. We noticed the following disposition in terms of binding efficacy from the highest to the lowest: grape seed meal > beetroot > white potato skins > red potato skins > seabuckthorn meal > carrot > Granny apple > celery. Similar to AFB1, the rate of ZEA adsorption indicates rapid binding (<5 min) and follows a steady trend up to 90 min of contact time, when the mycotoxin concentration becomes almost negligible with the increase in time. We noted significant changes in case of GSM and SBM, which registered the highest adsorption capacity (53.58% for GSM and 58.69% for SBM) at 24 h of incubation time. 

Despite the lack of isotherms to properly assess the parameters governing the adsorption kinetics, our results provide adequate information on the effect of incubation time on the binding process. We assume that the adsorption with respect to residence time on the surface of GSM and SBM shows that the removal percentage of the toxins is fast until they reached equilibrium. 

Moreover, considering the mycotoxin binding as binary-toxin adsorption, the difference in their rate of adsorption might be attributed to the by-product surface area accessibility, as well as to some degree of by-product specificity. In this context, given that the rate of adsorption decreases after reaching the plateau, the saturation of adsorbent surface might indicate different desorption intensities. That is to say, the rate of desorption might depend on the toxin [27,39]. In addition, as one of the purposes herein was to ascertain the effect of time on the capacity of different adsorbents to retain mycotoxins over a long period of time, these observations provide appropriate insight with respect to the comparative efficacy among the tested food by-products.

These findings suggest rapid binding potential, which may have important implications in reducing toxin bio-accessibility of the mycotoxins in the gastrointestinal (GI) tract [12,22], and merit further investigation. 

#### 2.3.2. Effect of Food by-Product Concentration 

**Figure 4:**
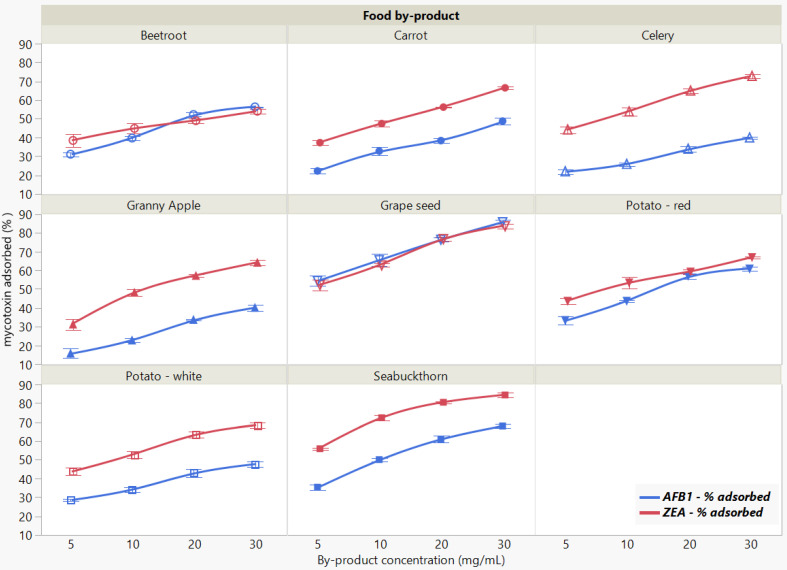
Effect of food by-product concentration on AFB1 and ZEA binding rates. Binding experiments were performed at constant pH (7), temperature (37 °C) and time (90 min), using 5 μg/mL mycotoxin concentration.

The binding of the two mycotoxins was significantly affected by food by-product concentration when measured at pH 7 (Figure 4). The total amount of removed mycotoxin increased with the increasing adsorbent concentration, revealing similar overall patterns for both AFB1 and ZEA adsorption. 

We observed a statistically significant interaction of food by-product by concentration (*p* < 0.0001 *), which is giving some indication that whatever food by-product effect (*p* < 0.0001 *) there is depends on concentration (*p* < 0.0001 *), qualifying the significant main effects in case of both mycotoxins. The comparative efficacy when increasing the concentration of evaluated by-products revealed that GSM and SBM exerted the highest sequestering potential for both AFB1 and ZEA. 

Batch adsorption experimental results are generally described by employing various models used for the study of adsorption mechanisms [40]. Langmuir and Freundlich are among the most common isotherm models used to characterize and contrast the potential of various biosorbents [41,42]. As opposed to the Langmuir model, which assumes the adsorption occurs at a definite number of localized sites, the Freundlich model provides information related to the equilibrium constant and the degree of heterogeneity, and cannot estimate the monolayer adsorption capacity [12,43]. 

By fitting the Freundlich isotherm model to the adsorption data, we estimated the Freundlich constant Kf and the heterogeneity index 1/n (Table 2).

The experimental values for K_f_ constant recorded at pH 7, which is associated with adsorbent affinity, reveal similar sequestering potential of GSM for both mycotoxins, whereas SBM displays higher affinity for ZEA. Correspondingly, the heterogeneity index 1/n reports on the binding sites associated with the adsorption process, suggesting homogeneity at values of 1/n = 1, and favourability at values of 1/n < 1. 

In this regard, the values (<1) estimated for the 1/n parameter render the binary-mycotoxin adsorption process favourable in case of both food by-products. Moreover, while GSM showed similar 1/n values for the two mycotoxins, SBM appears to exert a higher binding affinity towards ZEA, as implied by the 1/n parameter value (0.213) lower than that of AFB1 (0.341). As the experiment focuses on a binary-mycotoxin system, these findings serve as additional information to support the overall representation of comparative efficacy of the adsorption process.

#### 2.3.3. Effect of Medium pH

**Figure 5:**
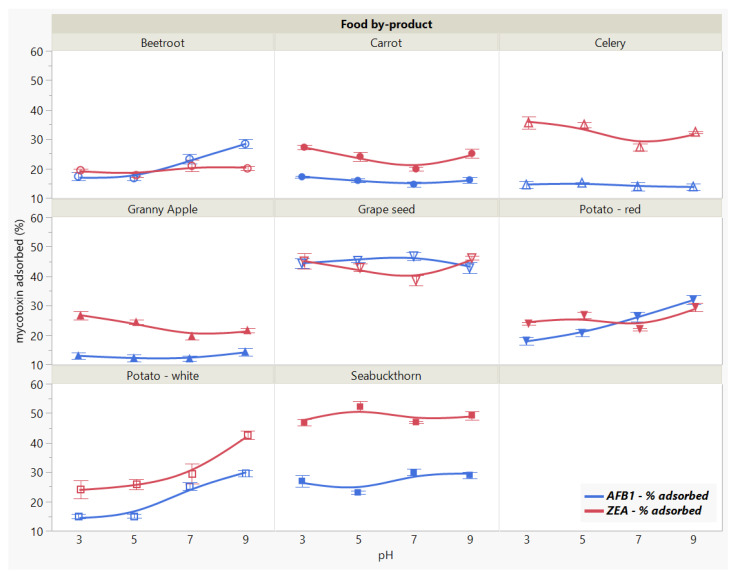
Effect of medium pH on AFB1 and ZEA binding rates. Binding experiments were performed at constant temperature (37 °C) and time (90 min), using 5 mg/mL food by-product concentration and 5 μg/mL mycotoxin concentration.

Similarly, the total amount of removed mycotoxin varied as the medium pH modified depending on the adsorbent (Figure 5). This was revealed for both AFB1 and ZEA by the significant main effects of food by-product (*p* < 0.0001 *) and pH (*p* < 0.0001 *), as well as by their interaction (*p* < 0.0001). Overall, the highest removal capacity for the two mycotoxins were obtained for GSM and SBM, showing slightly different patterns across the tested pH range. 

Apart from the patterns that resemble each other as a function of pH, we observed a specificity trend, suggesting the affinity of GSM towards AFB1 and of SBM towards ZEA. 

In general, the adsorption process is mitigated by the medium pH, as it affects the surface charge of the adsorbent along with the adsorbate degree of ionization [21,26]. Nonetheless, within the pH range of 3–9, AFB1 adsorption was constant for both food by-products, registering a slight increase at neutral pH in case of SBM. Similarly, pH did not significantly affect ZEA adsorption in the tested range. In case of GSM, ZEA recorded a lower bound amount at pH 7, whereas SBM absorbed more at pH 5 compared to the other assayed pH values. 

These observations suggest that AFB1 and ZEA adsorption onto GSM and SBM withstand the changes in pH throughout the gastrointestinal (GI) tract during digestion in monogastric animals, and are in agreement with previous findings [12]. This might be attributed to the presence and maintenance of strong bonds between the toxin molecules and the functional groups on the surface of the adsorbent [33,42]. 

#### 2.3.4. Effect of Incubation Temperature

**Figure 6:**
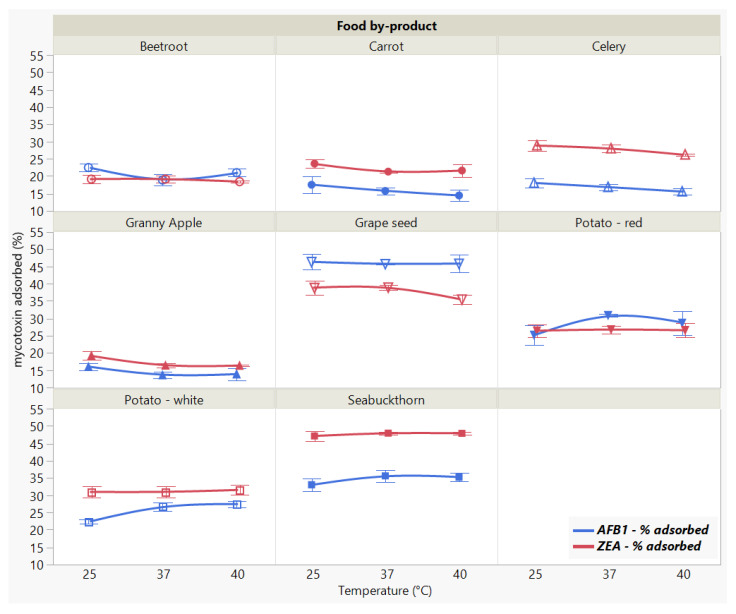
Effect of incubation temperature on AFB1 and ZEA binding rates. Binding experiments were performed at constant pH (7) and time (90 min), using 5 mg/mL food by-product concentration and 5 μg/mL mycotoxin concentration.

We did not observe a significant effect of the incubation temperature on the adsorption of ZEA (*p* < 0.8969), as opposed to the significant (*p* < 0.0001 *) differences registered for AFB1 (Figure 6). 

The different patterns displayed by the individual adsorbent materials (Food by-product, *p* < 0.0001 *, AFB1 and ZEA) weigh on the overall parallel trend across the temperature effect (Food by-product × Temperature, *p* < 0.0001 * for AFB1, and Food by-product × Temperature, *p* < 0.1865 for ZEA, respectively), suggesting that there is a different effect for tested food by-product at different temperatures in case of AFB1 reduction. At the same time, ZEA binding was not influenced by temperature variations. The results reveal GSM and SBM as the most effective in sequestering both AFB1 and ZEA. 

In actuality, the analysis of variance run only for the two materials indicated that temperature changes did not significantly affect mycotoxin uptake by GSM and SBM, which suggests the binding process is stable for both AFB1 and ZEA within the tested range. Generally, the influence of temperature is studied in order to assess the stability of the formed complexes in relation to the type of adsorption process [23]. In this regard, it is recommended that the tested temperature range includes a value of around 37 °C, as adsorbents are intended to exert their mode of action during their passing through the digestive tract [44]. Even though it might not provide sufficient information with regard to the thermodynamic parameters governing the adsorption process, the choice of temperature interval examined herein implies the target values associated with the animal body [12,23].

In order to avoid redundancy by duplicating the presentation of results, a complete depiction of the collective binding capacity is given as supplementary Information for each food by-product as follows: Supplementary Figures S12 and S13: Effect of incubation time on AFB1 and ZEA binding; Supplementary Figures S14 and S15: Food by-product concentration on AFB1 and ZEA binding; Supplementary Figures S16 and S17: Effect of medium pH on AFB1 and ZEA binding; Supplementary Figures S18 and S19: Effect of incubation temperature on AFB1 and ZEA binding. 

Further, the rationale was to ascertain the theoretical optimum conditions under which the binding capacity could be improved. After isolating GSM and SBM from the rest of the food by-products, we noted no effect of pH and temperature on the adsorption process, but incubation time and food by-product concentration showed significant differences on both AFB1 and ZEA sequestering potential (Table 3).

### 2.4. Response Surface Methodology Results

The developed process was destined to optimise the adsorption of mycotoxins by GSM and SBM, taking into consideration the critical parameters, including by-product concentration, incubation pH and temperature. Experimental and predicted values are presented in Supplementary Tables S1 and S2.

For the optimisation process, the incubation time was set to 24 h in order to ensure complete mycotoxin adsorption. We did not assess desorption kinetics, which could be perceived as a drawback. Nevertheless, taking into account the intended use of the by-products, which is their inclusion as supplements into animal diets, the 24 h period is relevant in terms of digestibility. 

Given the evaluated model was highly significant at a confidence interval of 95% (*p* < 0.0001 *), the correlation coefficients (R^2^) indicate a good fit to the experimental data (Table 4). Moreover, serving as a good descriptor of the explanatory power [45,46], R^2^ adjusted values are corrected according to the number of predictors in the model, and give an unbiased estimation [47,48]. 

Thus, our observations could provide supplementary information as to what extent the RSM model can explain the GSM and SBM potential pertaining the adsorption of the binary mycotoxin system. Having assumed proper fitting of the model, we noted a decreasing trend after computing the predicted R^2^ values, which might suggest model overfitting. In this context, the optimisation set-up presented in our study would require further examination.

**Figure 7:**
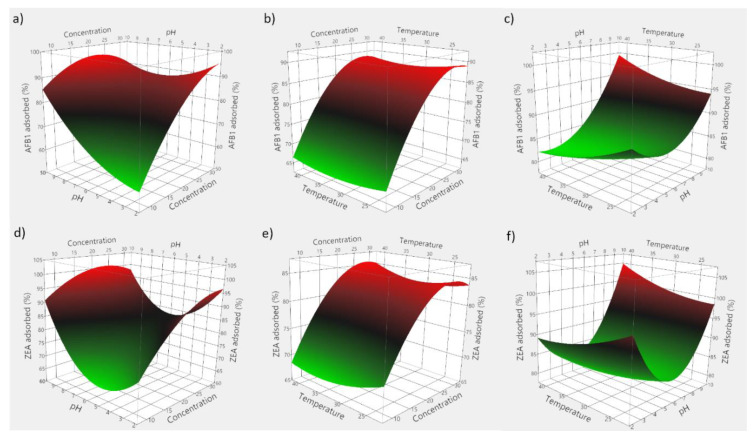
3D plots depicting the effect of the simultaneous variation of the adsorption process variables for GSM. (**a**) effect of food by-product concentration and medium pH on AFB1 adsorption; (**b**) effect of food by-product concentration and incubation temperature on AFB1 adsorption; (**c**) effect of medium pH and incubation temperature on AFB1 adsorption; (**d**) effect of food by-product concentration and medium pH on ZEA adsorption; (**e**) effect of food by-product concentration and incubation temperature on ZEA adsorption; (**f**) effect of medium pH and incubation temperature on ZEA adsorption.

**Figure 8:**
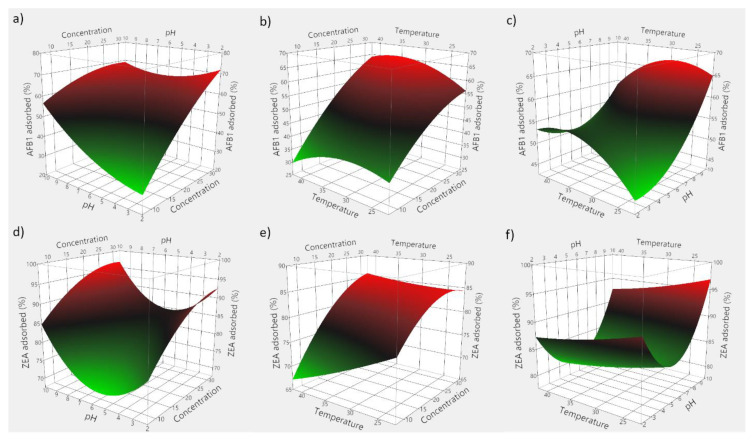
3D plots depicting the effect of the simultaneous variation of the adsorption process variables for SBM. (**a**) effect of food by-product concentration and medium pH on AFB1 adsorption; (**b**) effect of food by-product concentration and incubation temperature on AFB1 adsorption; (**c**) effect of medium pH and incubation temperature on AFB1 adsorption; (**d**) effect of food by-product concentration and medium pH on ZEA adsorption; (**e**) effect of food by-product concentration and incubation temperature on ZEA adsorption; (**f**) effect of medium pH and incubation temperature on ZEA adsorption.

Based on the obtained experimental data, 3D plots were constructed to visualize the predicted model. Figure 7 consists of 3D plots obtained for GSM, and depicts the effect of evaluated parameters on the adsorption of AFB1 (panels a–c) and ZEA (panels d–f). Correspondingly, Figure 8 displays the response surface plots revealing the effect of food by-product concentration, pH, and temperature on mycotoxin adsorption onto SBM.

The employed RSM model allowed for the computing of the optimal adsorption conditions, which were 29 mg/mL of GSM, pH 5.95 and 33.6 °C for AFB1, and 28 mg/mL of GSM, pH 5.76 and 31.7 °C for ZEA, respectively. Similarly, in case of SBM, the estimated optimal conditions of the adsorption process were 37.5 mg of adsorbent, pH 8.1 and 35.6 °C for AFB1, and 30.2 mg of adsorbent, pH 5.6 and 29.3 °C for ZEA, respectively. 

Food by-product concentration was shown to be highly significant (Table 4), and points to the importance of the proportion of solid material in achieving high adsorption of mycotoxins. Theoretically, higher adsorbent amounts would be necessary to attain improved mycotoxin binding, since a larger monolayer forms between the solid particles and the toxins [32,49]. This would entail higher population of binding sites and a corresponding increased adsorption intensity [12]. 

Similarly, we observed a significant effect of pH on the adsorption process in all instances. On the other hand, the negative food by-product concentration and pH interaction effect would suggest that elevated pH might unfavorably interact with increased by-product concentration. Hence, improved mycotoxin binding may be achieved at low pH values. Such an effect would be manifested during digestion as the supplemented feed passes through the GI compartments where pH changes occur [12,33]. On the other hand, the quadratic positive effect of pH might suggest that ZEA adsorption by both agricultural by-products would increase both at low and high pH values. 

As a way of profiling the differences in the two-way interaction, this might be attributed to the shift in the equilibrium towards adsorption of mycotoxins onto GSM and SBM, with reduced saturation of mycotoxin concentration [41,50,51]. However, these findings encourage further examination with regard to the adsorbent surface charge and the different degrees of ionization of the involved molecules.

Regarding the temperature influence, earlier investigations showed that 11 mycotoxins, including AFB1 and ZEA, were predicted to display maximum adsorption at a temperature of 35 °C using chitosan mediated removal from palm kernel cake [22]. In the case herein, the positive interaction effect between food by-product concentration and temperature appears to be significant only in case of ZEA adsorption onto SBM, and indicates that improved binding could occur by increasing both the amount of seabuckthorn meal as well as the temperature. Similarly, Abbasi Pirouz et al. (2018) suggested that the increase of temperature from 30 to 50 °C would cause an increase in the adsorption of AFB1, OTA, and ZEA onto magnetic graphene oxide modified with chitosan [42]. However, the insignificant quadratic effect of incubation temperature might set a limitation to the extent to which it could be increased in order to attain favourable or improved binding (Figure 8e). 

Envisaging the food by-products inclusion in animal feed, the model described herein suggests that the factors influencing mycotoxin binding in vivo would be adsorbent concentration and medium pH. Accordingly, not to disregard the potential negative effects, grape seeds and seabuckthorn have been ascribed to possess beneficial properties in both humans and animals [14,15,52,53]. Moreover, while it is generally assumed that the involved mechanism is physisorption [54,55], the adsorption process is also influenced by the chemical properties and interactions between the adsorbents and mycotoxins [54,56]. Similar studies reported the importance of surface conformation and chemical composition in relation to the interactions between adsorbent functional groups and mycotoxin molecules under acidic conditions [27,33,56].

In this context, it could be argued that, besides their main utility as adsorbents, the food by-products could also be perceived as nutritive additives [56,57].

Taking into account the adsorption percentages and the predicted adsorption favourable settings obtained within the physiological range, we believe the evaluated by-products show promising potential with regard to their inclusion into animal feed as adsorbent material.

## 3. Conclusions

The present work presented the ability of eight food by-products to be used as low-cost biosorbents for the removal of mycotoxins from liquid media. Overall, the binding process was highly dependent on the adsorbent. The comparative efficacy among the tested food by-products revealed varying disposition of the food by-products in terms of binding capacity, with grape seed and seabuckthorn meals exerting the highest sequestering potential for both AFB1 and ZEA. The assessment of critical parameters highlighted not only that the adsorption process occurs rapidly, but also in a concentration dependent manner. This is highly relevant with regard to the significant influence of adsorbent concentration on mycotoxin sequestering. In addition, judging from the perspective of their inclusion rates in animal diets, the interposing specificity of GSM and SBM towards the binary-mycotoxin system might be regarded as highly important. These findings on the comparative efficacy of GSM and SBM to bind AFB1 and ZEA merit further investigation, as their application would be advantageous in terms of added-value functionality (bio-adsorption, feed supplementation), as well as agricultural and food waste reduction. The by-products with the best results, grape seed and seabuckthorn meals have already been investigated in a first in vivo trial on pigs in our experimental farm. The preliminary results showed that the inclusion of 5% mix of the two of by-products (1:1) was able to reduce the concentration of mycotoxin in liver and kidney. Other rates of inclusion will be further investigated in order to determine the most effective concentration.

## 4. Materials and Methods

### 4.1. Reagents and Chemicals

Aflatoxin B1 (AFB1) and Zearalenone (ZEA), both of analytical grade, were obtained from Fermentek Ltd., Jerusalem, Israel. Phosphate buffered saline (PBS) was obtained from Sigma-Aldrich, Darmstadt, Germany. All solvents used for chromatography were of HPLC analysis grade. All other reagents were of analytical purity or chromatographic grade. The ultra-pure water was obtained using a water purification system (Barnstead, EASYPURE II, ThermoScientific, Waltham, MA, USA). All solutions were stored at 4 °C and protected from light, and were filtered before analysis using nonsterile micro-centrifugal filters 0.2 μm (regenerated cellulose, Thermo Fisher Scientific, Waltham, MA, USA).

The solid toxins (AFB1 and ZEA) were dissolved in acetonitrile (HPLC grade) to obtain stock solutions of AFB1 and ZEA (1 mg/mL), which were stored in the dark at 4 °C. A binary-mycotoxin standard solution containing 100 μg/mL of each toxin (AFB1 and ZEA) was prepared by mixing equal volumes of mycotoxin stock solutions. Subsequently, this solution was appropriately diluted with PBS buffer at different pH values to prepare the mycotoxin working solutions for binding experiments. The mycotoxin stock solution was separately appropriately diluted with acetonitrile in order to prepare the standard samples for UHPLC calibration curves in the range of 0.05–6.25 μg/mL.

### 4.2. Sample Preparation

Eight food by-products were used in the present study. Grape seed and seabuckthorn meals were received from local distributors. White and red skin potatoes were manually pealed, while Granny apple, carrot, beetroot and celery were processed under conventional household conditions for juice making. Briefly, each fruit or vegetable product was chopped and inserted into a common commercial juicer in order to extract the juice. The juice moves through a tube and into a pitcher, while the pulp and peel are left behind. Subsequently, the resulting food by-products were freeze-dried using a LyoQuest freeze-dryer (Azbil Telstar, S.L.U., Terrassa, Spain) and ground into a fine powder. They were further investigated for AFB1 and ZEA initial contamination as previously reported by Taranu et al. (2019) and showed the absence of mycotoxins [58]. The analysis was performed by ELISA method using Veratox ELISA kits (Neogen, Lansing, MI, USA) according to the manufacturer’s instructions, with the limit of detection (LOD) of 5 × 10 ^−7^ mg/mL for AFB1 and 5 × 10 ^−6^ mg/mL for ZEA, and the limit of quantification (LOQ) of 1–8 × 10 ^−6^ mg/mL for AFB1 and 25–500 × 10 ^−6^ mg/mL for ZEA, respectively.

### 4.3. Experimental Design

Mycotoxin binding experiments were conducted according to the method described by Avantaggiato et al. [12], who used adsorption models to explain the reduction of mycotoxin concentration. Briefly, the food by-products were weighed in Eppendorf tubes, suspended with an appropriate volume of binary-mycotoxin working solution in PBS buffer, vortexed vigorously, incubated at constant temperature in a thermostatically controlled shaker at 250 rpm, and finally centrifuged. The supernatant was diluted by mixing 700 μL of the sample (in PBS) with 300 μL of acetonitrile/methanol (1:2, *v*/*v*) mixture containing 1% acetic acid, then filtered using nonsterile micro-centrifugal filters 0.2 μm RC (Thermo Fisher Scientific, USA), and analysed for the residual mycotoxin content by UHPLC. Mycotoxin working solutions in buffer without food by-products represented background controls for the adsorption experiments.

#### 4.3.1. In Vitro Binding Experiments

In order to assess the in vitro mycotoxin binding efficacy of the food by-products, preliminary evaluation was performed to determine the effect of target parameters (contact time, food by-product concentration, medium pH, and temperature) on the reduction of mycotoxins in liquid media. 

The following describes the parameters: contact time was the incubation period in minutes; food by-product concentration was the amount of freeze-dried food by-product added in the mixture of the liquid media (mg/mL); medium pH was the pH in the model solution where the adsorption experiments occur; temperature was the temperature (in degrees Celsius) of the incubation environment within the thermostatically controlled shaker. 

Mycotoxin concentration was 5 μg/mL in all cases. The analysis was conducted in three individual experiments, in which the samples were analysed in duplicate. Table 5 summarizes the parameter values and experimental setup employed in order to study the effect of incubation time, food by-product concentration, medium pH, and temperature on the adsorption process.

To study the effect of incubation time on the adsorption process, the food by-products (0.5% *w*/*v* (5 mg/mL)) were tested at pH 7, 37 °C, 250 rpm. Samples were collected at appropriate time intervals (1 min–24 h).

To investigate the effect of food by-product concentration on mycotoxin adsorption, the experiments were performed at pH 7, 90 min, 37 °C, 250 rpm, testing different amounts of by-product (residue) (0.5–3% *w*/*v* corresponding to 5–30 mg/mL). 

To study the effect of medium pH, samples were assessed at 0.5% *w*/*v* food by-products (5 mg/mL), 90 min, 37 °C, 250 rpm and variable pH values (pH 3, 5, 7, and 9).

The effect of temperature was investigated by testing a fixed amount of food by-product 0.5% *w*/*v* (5 mg/mL) at pH 7, for 90 min at 250 rpm at different temperature (25, 37, and 40 °C).

#### 4.3.2. Evaluation of Adsorption Using Response Surface Methodology (RSM)

In view of assessing the influence of critical parameters affecting adsorption efficacy, an experimental design was set up including food by-product concentration, medium pH and temperature, while keeping incubation time constant at 24 h. 

The chosen approach was a central composite design (CCD), employing a response surface methodology (RSM) screening output, composed in total of 32 runs (design points). The percentage of mycotoxin adsorption was the screening response, and the three independent variables (factors) were food by-product concentration, medium pH, and temperature, which were coded from “-” (lower limit) to “+” (upper limit), with two center points (coded “0”), two axial points (coded “a” and “A”) and two replicates. Table 6 summarizes the information about the factors and their settings. Surface 3D plots were constructed to visualize the predicted model using SAS JMP™ (SAS Analytics, USA) software.

### 4.4. UHPLC Analysis

The method development, including the selection of solvents for the mobile phase, was done based on previously reported chromatographic methods as model [12,32,34,59]. Several experiments were carried out to achieve an appropriate resolution, as well as a better signal to noise ratio for detection. In order to optimise the total analysis time, the following parameters were successively varied: mobile phase, flow rate and column temperature. We studied their influence on the retention time values. Five different solvent gradients, 4 flow rate variations and 4 column temperature settings were tested to ensure proper resolved peaks. 

The determination of mycotoxins was performed using an UltiMate™ 3000 UHPLC system (Thermo Scientific TM), equipped with a UltiMate™ LPG-3400SD quaternary pump, a UltiMate™ ACC-3000 autosampler with integrated column compartment, an UltiMate™ DAD-3000 UV-Vis detector, an UltiMate™ FLD-3100 fluorescence detector, and a Chromeleon 7 spectral analysis module. 

The chromatographic separation of mycotoxins was done on an Acclaim™ 120 C18 (150 × 4.6 mm, 5 µm particle size) column, for 12 min at 40 °C (column temperature), using isocratic elution (A/B/C—50/30/20%) with 0.1% formic acid in water (solvent A), 0.1% formic acid in methanol (solvent B), and acetonitrile (solvent C). The injected volume was 10 μL in a partial loop with needle over-fill mode. The flow rate was as follows: 0–5 min 0.5 mL/min; 5.01–10 min 0.6 mL/min; 10.01–12 min 0.5 mL/min. The column was equilibrated for 30 min prior to initiating injections. Fluorescence monitoring and assessment of mycotoxins was achieved using an excitation wavelength of 270 nm and an emission wavelength of 440 nm. The DAD detector was used for the initial steps of method development, employing tandem monitoring at four different wavelengths, of which only two channels were kept for further evaluation (280 nm and 362 nm). All the analyses were performed in triplicate.

### 4.5. Method Validation

In order to verify the performance characteristics of the (developed) UHPLC method, we evaluated the following parameters: limit of detection and limit of quantification, selectivity, accuracy, linearity, and precision (repeatability and reproducibility) [59,60].

LOD and LOQ were evaluated on 5 consecutive determinations of a standard sample of 0.05 μg/mL, and were calculated based on the signal-to-noise ratio (S/N) of 3 and 10 for LOD and LOQ, respectively. Selectivity was studied on a standard mixture containing 5 μg/mL of AFB1 and ZEA. In order to estimate accuracy, we performed 5 repeated determinations of blank matrix samples spiked with known concentrations of AFB1 and ZEA (5 μg/mL). Linearity was determined by running seven-level concentrations prepared from the binary-mycotoxin stock solution, over the range of 0.05–6.25 μg/mL for both AFB1 and ZEA, respectively. Method repeatability (intra-day assay) was evaluated by calculating the RSD for 5 repeated determinations run on 5 samples containing 0.5 μg/mL, 1.5 μg/mL, 2.5 μg/mL, 5 μg/mL and 6.25 μg/mL standard mixture of AFB1 and ZEA. Method reproducibility (inter-day assay) was evaluated by analysing standard mixture of 5 μg/mL by two different analysts on five consecutive days, and was reported as RSD.

### 4.6. Data Calculation and Statistical Analysis

The quantity of bound mycotoxin was calculated as the difference between the mycotoxin amount in the background controls and the residual mycotoxin amount in the sample tubes. Adsorption data were expressed as percentage of mycotoxin adsorbed and plotted according to the analysed parameter.

The estimation of the maximum adsorption capacity requires the variation of the initial concentration of the substance that is adsorbed. For convenience, the sorbent dose is generally optimised before isotherm experimentation. In this context, the variation of initial mycotoxin concentration was not included in the experimental design, rendering the application of any isotherm models inconclusive and biased. Nonetheless, within the present study we operated with constant initial concentration of mycotoxins and variable weights of adsorbent (5, 10, 20, and 30 mg). These experimental points were used to calculate the adsorption affinity and heterogeneity of the adsorption process, associated with the adsorption of AFB1 and ZEA onto GSM and SBM (Table 2). For future comparison purposes, model goodness-of-fit estimates include the error sum of squares (SSE), the error degrees of freedom (DFE), the mean square error (MSE), and the root mean square error (RMSE). Reporting the R^2^ is based on the underlying assumption of fitting a linear model, and calculating this statistic for nonlinear regression is a dubious practice that produces bad outcomes [61]. Accordingly, we reported the corrected Akaike information criterion (AICc), which is an appropriate statistical tool for ranking of adsorption isotherm models [62].

The Freundlich isotherm model was fitted to the adsorption data, and allowed the estimation of the Freundlich constant K_f_ and the heterogeneity index 1/n using Equation (1):

Q_eq_ = K_f_C_eq_^1/n^ (1)

where Q_eq_ is the amount of adsorbed mycotoxin per amount of food by-product (μg/mg), K_f_ is the Freundlich constant related to the adsorbent capacity towards the mycotoxin, C_eq_ is the residual mycotoxin concentration in the sample tubes at equilibrium (μg/mL), and n is the adsorption intensity.

All data are expressed as mean ± standard deviation. All the results were submitted to SAS JMP™ software and R Project (R Foundation for Statistical Computing, Vienna, Austria) software. Analysis of variance (ANOVA), employing repeated measurements analysis, was performed to investigate the statistical differences between groups for all analysed parameters. The response surface methodology (RSM) approach was employed to estimate favourable adsorption conditions. All significance levels were established at a 95% level (*p* < 0.05).

## Figures and Tables

**Table 1:** Results obtained for the validation of the UHPLC method for the determination of aflatoxin B1 and zearalenone.

Parameters	AFB1	ZEA
Precision		
Repeatability, RSD	1.58	1.61
Reproducibility, RSD	2.41	2.83
Accuracy		
Accuracy	100.76	101.64
bias	0.76	1.64
Limits of Detection and Quantification-Sensitivity		
LOD (μg/mL)	0.15	0.15
LOQ (μg/mL)	0.50	0.50
Linearity		
Domain (μg/mL)	0.05	6.25
R^2^ value	0.998	0.998
RSD	5.37	5.55

**Table 2:** Freundlich isotherm model parameters for the adsorption of mycotoxins by GSM and SBM at pH 7.

**GSM**							
	**K_f_**	**1/n**	**SSE**	**DFE**	**MSE**	**RMSE**	**AICc**
AFB1	0.367	0.248	1.08 × 10 ^−4^	2	5.414 × 10 ^−5^	7.35 × 10 ^−3^	−118.387
ZEA	0.343	0.264	1.64 × 10 ^−4^	2	8.233 × 10 ^−5^	9.07 × 10 ^−3^	−113.357
**SBM**							
	**K_f_**	**1/n**	**SSE**	**DFE**	**MSE**	**RMSE**	**AICc**
AFB1	0.217	0.341	1.31 × 10 ^−3^	2	6.56 × 1 ^−4^	0.025	−88.445
ZEA	0.418	0.213	3.07 × 10 ^−3^	2	1.53 × 10 ^−3^	0.039	−78.234

SSE = error sum of squares; DFE = error degrees of freedom; MSE = mean square error; RMSE = root mean square error; AICc = corrected Akaike information criterion.

**Table 3:** Influence of adsorption parameters on GSM and SBM.

Term	*p* Value	
	*AFB1*	*ZEA*
Food by-product	<0.0001 *	<0.0001 *
Contact time (min)	<0.0001 *	<0.0001 *
Food by-product concentration (mg/mL)	<0.0001 *	<0.0001 *
Medium pH	0.8210	0.8342
Temperature (°C)	0.0739	0.3796

* significant effect (α = 0.05).

**Table 4:** Estimates of model coefficients and effects of the RSM model showing significance of evaluated factors.

Term	Parameter Estimates				*p* Value			
	GSM		SBM		GSM		SBM	
	*AFB1*	*ZEA*	*AFB1*	*ZEA*	*AFB1*	*ZEA*	*AFB1*	*ZEA*
Food by-product concentration (10,30)	8.681	5.901	11.108	5.305	<0.0001 *	<0.0001 *	<0.0001 *	<0.0001 *
pH (3,9)	4.263	3.369	4.612	2.037	0.0010 *	0.0023 *	0.0083 *	0.0044 *
Temperature (25,40)	−0.190	−0.112	0.003	−1.535	0.8665	0.9098	0.9981	0.0258 *
Food by-product concentration × pH	−4.883	−2.313	−4.578	−0.383	0.0008 *	0.0458 *	0.0173 *	0.5986
Food by-product concentration × Temperature	−0.006	0.600	2.068	1.724	0.9961	0.5884	0.2573	0.0252 *
pH × Temperature	1.744	1.970	−1.932	−0.004	0.1770	0.0852	0.2891	0.9953
Food by-product concentration × Food by-product concentration	−4.851	−3.741	−2.499	−2.270	0.0365 *	0.0621	0.4285	0.0831
pH × pH	3.373	8.102	3.002	5.592	0.1358	0.0003 *	0.3430	0.0002 *
Temperature × Temperature	0.759	0.955	−2.726	0.265	0.7308	0.6208	0.3884	0.8336
**Goodness-of-fit**	*p* value	RSquare	RMSE	RSquare Adj.	PRESS	PRESS RMSE	Predicted RSquare	
**GSM**								
AFB1	<0.0001 *	0.82	5.01	0.74	1337.82	6.46	0.55	
ZEA	<0.0001 *	0.78	4.37	0.69	1007.13	5.61	0.48	
**SBM**								
AFB1	<0.0001 *	0.76	7.11	0.66	2400.11	8.66	0.48	
ZEA	<0.0001 *	0.84	2.87	0.77	389.14	3.49	0.65	

* significant effect (α = 0.05).

**Table 5:** Tested parameter values and setup of adsorption process.

Parameter	Tested Interval	Fixed Conditions
Time (min)	1 min–24 h	pH 7, 0.5% *w*/*v* (5 mg/mL), 37 °C, 250 rpm
Food by-product concentration (mg/mL)	0.5–3% *w*/*v* (5–30 mg/mL)	pH 7, 90 min, 37 °C, 250 rpm
pH	pH 3, 5, 7 and 9	0.5% *w*/*v* (5 mg/mL), 90 min, 37 °C, 250 rpm
Temperature (°C)	25 °C, 37 °C and 40 °C	0.5% *w*/*v* (5 mg/mL) at pH 7, for 90 min at 250 rpm

**Table 6:** Values and coded levels of factors.

Factors	Coded Factor Level				
	–	a	0	A	+
Concentration (mg/mL)	10	10	20	30	30
pH	3	3	6	9	9
Temperature (°C)	25	25	32.5	40	40

## Supplementary Materials

The following are available online at https://www.mdpi.com/2072-6651/13/1/2/s1, Figures S1–S11: Example chromatograms for UHPLC method optimisation; Figures S12 and S13: Effect of incubation time on AFB1 and ZEA binding; Figures S14 and S15: food by-product concentration on AFB1 and ZEA binding; Figures S16 and S17: Effect of medium pH on AFB1 and ZEA binding; Figures S18 and S19: Effect of incubation temperature on AFB1 and ZEA binding; Table S1: Experimental runs (design points) and corresponding measured and predicted adsorption values (%)—Grape seed meal; Table S2: Experimental runs (design points) and corresponding measured and predicted adsorption values (%)—Seabuckthorn meal.
