# Supplementary material for: Assessment of Food By-Products’ Potential for Simultaneous Binding of Aflatoxin B1 and Zearalenone
Source: Toxins (Basel). 2020 Dec 22;13(1):2. doi: 10.3390/toxins13010002 (PMC7822050; doi:10.3390/toxins13010002)
Supplement: Supplementary file 1 [file toxins-13-00002-s001.zip › Table S1.docx]

**Table S1**. Experimental runs (design points) and corresponding measured and predicted adsorption values (%) – Grape seed meal

| **Runs** | **Pattern** | **Factors** | | | **Response (AFB1 - % adsorbed)** | | **Response (ZEA - % adsorbed)** | |
| --- | --- | --- | --- | --- | --- | --- | --- | --- |
|  |  | Residue dosage (mg) | pH | Temp. (°C) | Measured | Predicted | Measured | Predicted |
| 1 | ++- | + | + | - | 94.136 | 94.242 | 89.663 | 91.012 |
| 2 | +-+ | + | - | + | 92.612 | 91.05 | 90.509 | 89.877 |
| 3 | 000 | 0 | 0 | 0 | 79.534 | 77.7 | 83.867 | 81.198 |
| 4 | a00 | a | 0 | 0 | 69.678 | 68.47 | 70.335 | 71.555 |
| 5 | 0a0 | 0 | a | 0 | 88.09 | 88.036 | 82.977 | 85.931 |
| 6 | 000 | 0 | 0 | 0 | 86.816 | 84.804 | 83.867 | 81.198 |
| 7 | 00A | 0 | 0 | A | 85.124 | 83.246 | 84.436 | 82.042 |
| 8 | +-- | + | - | - | 91.868 | 91.09 | 94.390 | 92.841 |
| 9 | --+ | - | - | + | 52.246 | 62.362 | 63.392 | 72.247 |
| 10 | -+- | - | + | - | 79.014 | 83.766 | 82.055 | 85.038 |
| 11 | +-- | + | - | - | 90.156 | 89.082 | 94.390 | 92.841 |
| 12 | 0a0 | 0 | a | 0 | 85.422 | 86.382 | 82.977 | 85.931 |
| 13 | 0A0 | 0 | A | 0 | 87.428 | 90.958 | 91.505 | 92.670 |
| 14 | 000 | 0 | 0 | 0 | 81.388 | 78.752 | 83.867 | 81.198 |
| 15 | --- | - | - | - | 71.926 | 82.21 | 67.249 | 77.613 |
| 16 | A00 | A | 0 | 0 | 89.9 | 87.594 | 87.697 | 83.357 |
| 17 | +++ | + | + | + | 92.098 | 93.902 | 92.759 | 95.929 |
| 18 | +-+ | + | - | + | 91.616 | 91.24 | 90.509 | 89.877 |
| 19 | 00A | 0 | 0 | A | 82.936 | 81.806 | 84.436 | 82.042 |
| 20 | 00a | 0 | 0 | A | 84.08 | 79.948 | 84.817 | 82.266 |
| 21 | A00 | A | 0 | 0 | 84.538 | 81.562 | 87.697 | 83.357 |
| 22 | 00a | 0 | 0 | A | 85.374 | 81.698 | 84.817 | 82.266 |
| 23 | 000 | 0 | 0 | 0 | 89.722 | 87.378 | 83.867 | 81.198 |
| 24 | -+- | - | + | - | 82.136 | 84.256 | 82.055 | 85.038 |
| 25 | --- | - | - | - | 65.394 | 78.214 | 67.249 | 77.613 |
| 26 | +++ | + | + | + | 90.848 | 93.24 | 92.759 | 95.929 |
| 27 | -++ | - | + | + | 80.558 | 84.044 | 85.175 | 87.554 |
| 28 | -++ | - | + | + | 96.81 | 97.056 | 85.175 | 87.554 |
| 29 | 0A0 | 0 | A | 0 | 87.032 | 89.908 | 91.505 | 92.670 |
| 30 | a00 | a | 0 | 0 | 70.956 | 70.282 | 70.335 | 71.555 |
| 31 | ++- | + | + | - | 92.268 | 93.038 | 89.663 | 91.012 |
| 32 | --+ | - | - | + | 67.71 | 77.358 | 63.392 | 72.247 |

For codified variable assignment, see Table 1.
