# Supplementary material for: Assessment of Food By-Products’ Potential for Simultaneous Binding of Aflatoxin B1 and Zearalenone
Source: Toxins (Basel). 2020 Dec 22;13(1):2. doi: 10.3390/toxins13010002 (PMC7822050; doi:10.3390/toxins13010002)
Supplement: Supplementary file 1 [file toxins-13-00002-s001.zip › Table S2.docx]

**Table S2**. Experimental runs (design points) and corresponding measured and predicted adsorption values (%) – Seabuckthorn meal

| **Runs** | **Pattern** | **Factors** | | | **Response (AFB1 - % adsorbed)** | | **Response (ZEA - % adsorbed)** | |
| --- | --- | --- | --- | --- | --- | --- | --- | --- |
|  |  | Residue dosage (mg) | pH | Temp. (°C) | Measured | Predicted | Measured | Predicted |
| 1 | ++- | + | + | - | 67.598 | 93.64 | 63.876 | 91.923 |
| 2 | +-+ | + | - | + | 70.698 | 90.27 | 67.953 | 88.993 |
| 3 | 000 | 0 | 0 | 0 | 55.79 | 83.516 | 55.097 | 81.560 |
| 4 | a00 | a | 0 | 0 | 61.944 | 80.86 | 41.490 | 73.984 |
| 5 | 0a0 | 0 | a | 0 | 55.274 | 84.904 | 53.488 | 85.116 |
| 6 | 000 | 0 | 0 | 0 | 60 | 83.444 | 55.097 | 81.560 |
| 7 | 00A | 0 | 0 | A | 56.058 | 80.914 | 52.375 | 80.291 |
| 8 | +-- | + | - | - | 55.552 | 85.56 | 59.944 | 88.607 |
| 9 | --+ | - | - | + | 26.358 | 74.032 | 32.443 | 74.167 |
| 10 | -+- | - | + | - | 48.378 | 84.6 | 54.953 | 85.527 |
| 11 | +-- | + | - | - | 66.866 | 90.852 | 59.944 | 88.607 |
| 12 | 0a0 | 0 | a | 0 | 57.588 | 84.108 | 53.488 | 85.116 |
| 13 | 0A0 | 0 | A | 0 | 60.668 | 88.698 | 62.712 | 89.190 |
| 14 | 000 | 0 | 0 | 0 | 51.014 | 82.002 | 55.097 | 81.560 |
| 15 | --- | - | - | - | 41.06 | 82.734 | 32.708 | 80.677 |
| 16 | A00 | A | 0 | 0 | 58.29 | 83.952 | 63.706 | 84.595 |
| 17 | +++ | + | + | + | 65.406 | 91.618 | 64.156 | 92.292 |
| 18 | +-+ | + | - | + | 67.618 | 89.27 | 67.953 | 88.993 |
| 19 | 00A | 0 | 0 | A | 54.266 | 80.84 | 52.375 | 80.291 |
| 20 | 00a | 0 | 0 | A | 50.986 | 81.804 | 52.367 | 83.361 |
| 21 | A00 | A | 0 | 0 | 54.858 | 84.336 | 63.706 | 84.595 |
| 22 | 00a | 0 | 0 | A | 51.174 | 82.502 | 52.367 | 83.361 |
| 23 | 000 | 0 | 0 | 0 | 47.59 | 79.774 | 55.097 | 81.561 |
| 24 | -+- | - | + | - | 58.37 | 85.214 | 54.953 | 85.527 |
| 25 | --- | - | - | - | 20.922 | 80.932 | 32.708 | 80.677 |
| 26 | +++ | + | + | + | 65.592 | 90.968 | 64.156 | 92.292 |
| 27 | -++ | - | + | + | 41.714 | 75.36 | 46.959 | 79.001 |
| 28 | -++ | - | + | + | 48.926 | 83.756 | 46.959 | 79.001 |
| 29 | 0A0 | 0 | A | 0 | 61.87 | 89.658 | 62.712 | 89.191 |
| 30 | a00 | a | 0 | 0 | 38.3 | 66.766 | 41.490 | 73.984 |
| 31 | ++- | + | + | - | 66.794 | 92.358 | 63.876 | 91.923 |
| 32 | --+ | - | - | + | 31.14 | 72.464 | 32.443 | 74.167 |

For codified variable assignment, see Table 1.
