# Supplementary figures and images for: Assessment of Food By-Products’ Potential for Simultaneous Binding of Aflatoxin B1 and Zearalenone
Source: Toxins (Basel). 2020 Dec 22;13(1):2. doi: 10.3390/toxins13010002 (PMC7822050; doi:10.3390/toxins13010002)

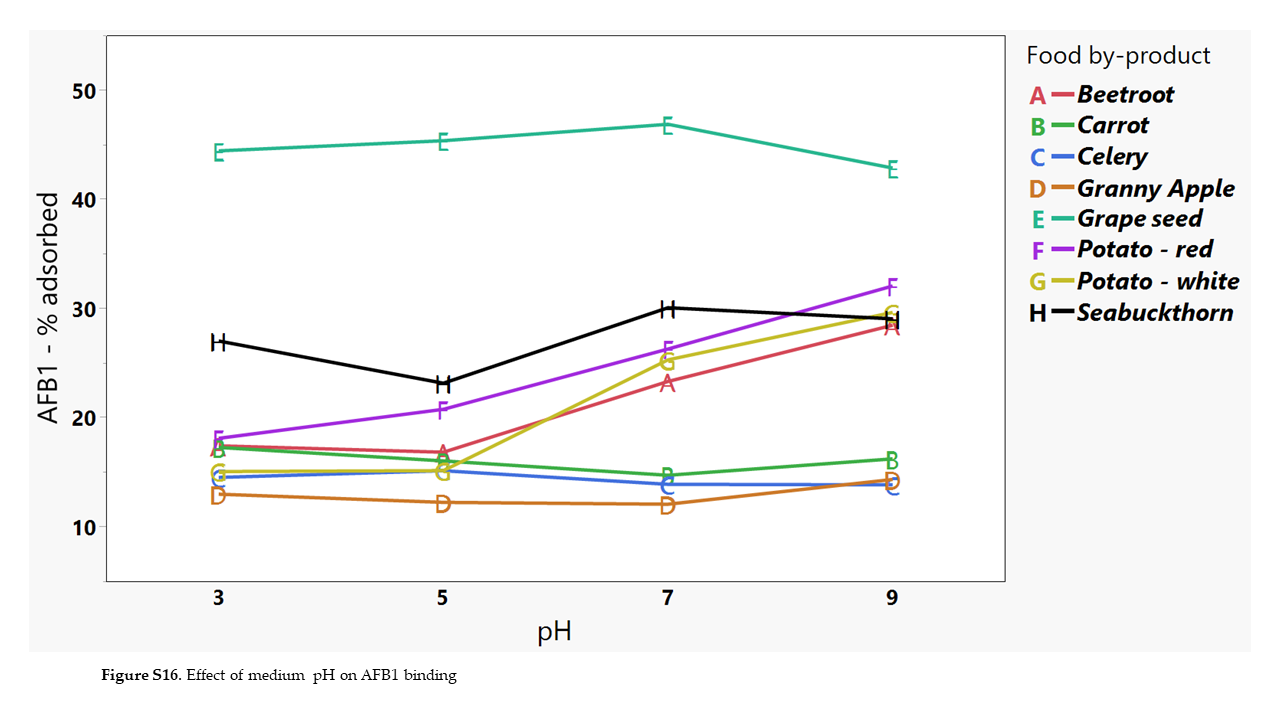

Supplement: Supplementary file 1 [file toxins-13-00002-s001.zip › Figures S16.tif]

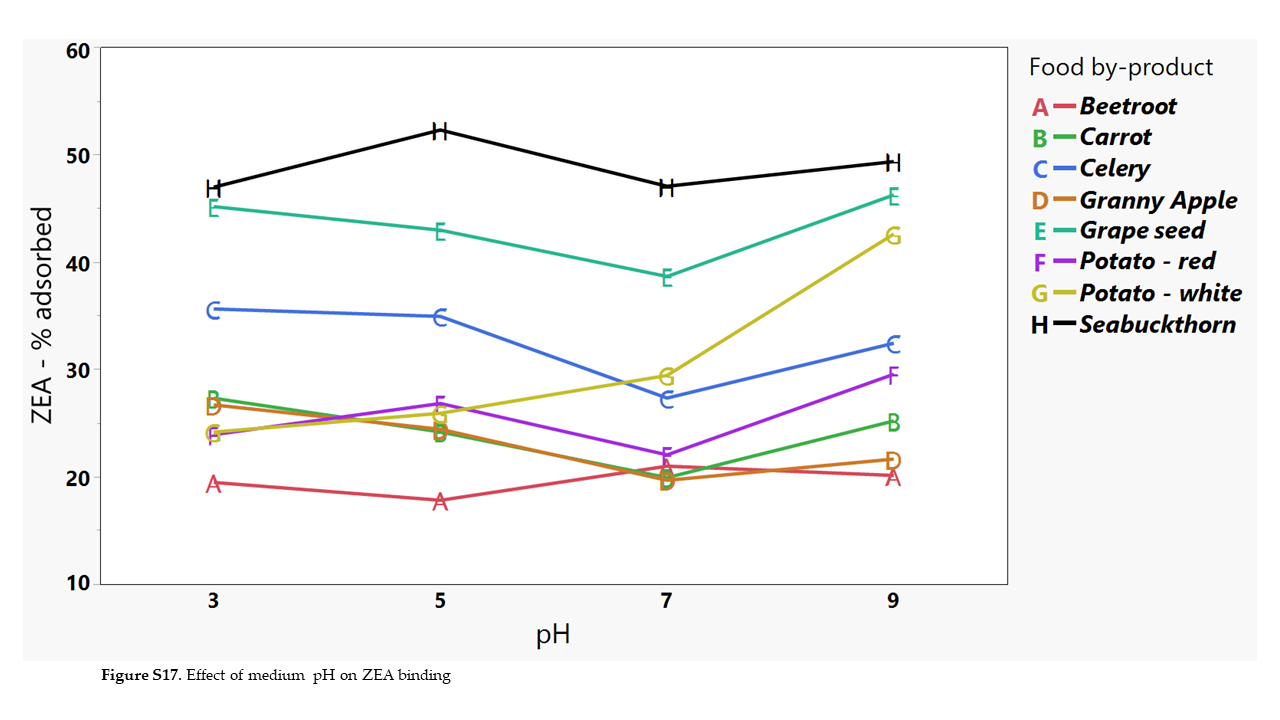

Supplement: Supplementary file 1 [file toxins-13-00002-s001.zip › Figures S17.tif]

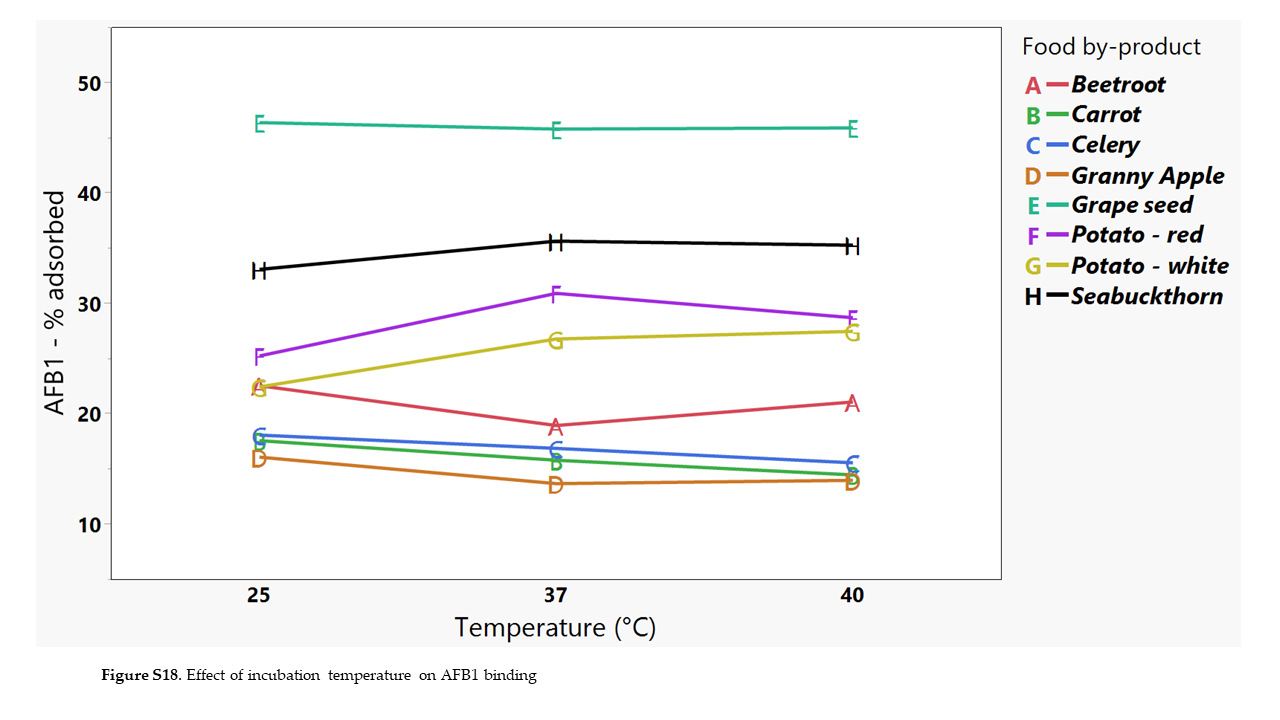

Supplement: Supplementary file 1 [file toxins-13-00002-s001.zip › Figures S18.tif]

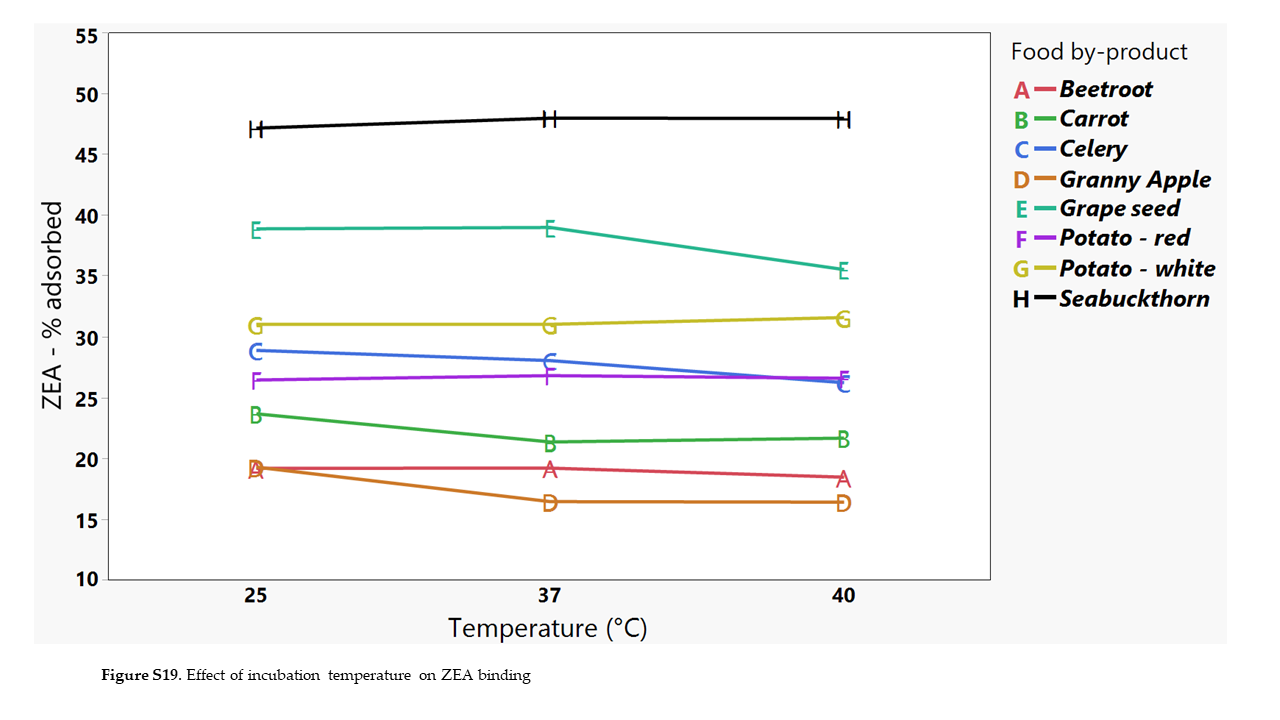

Supplement: Supplementary file 1 [file toxins-13-00002-s001.zip › Figures S19.tif]

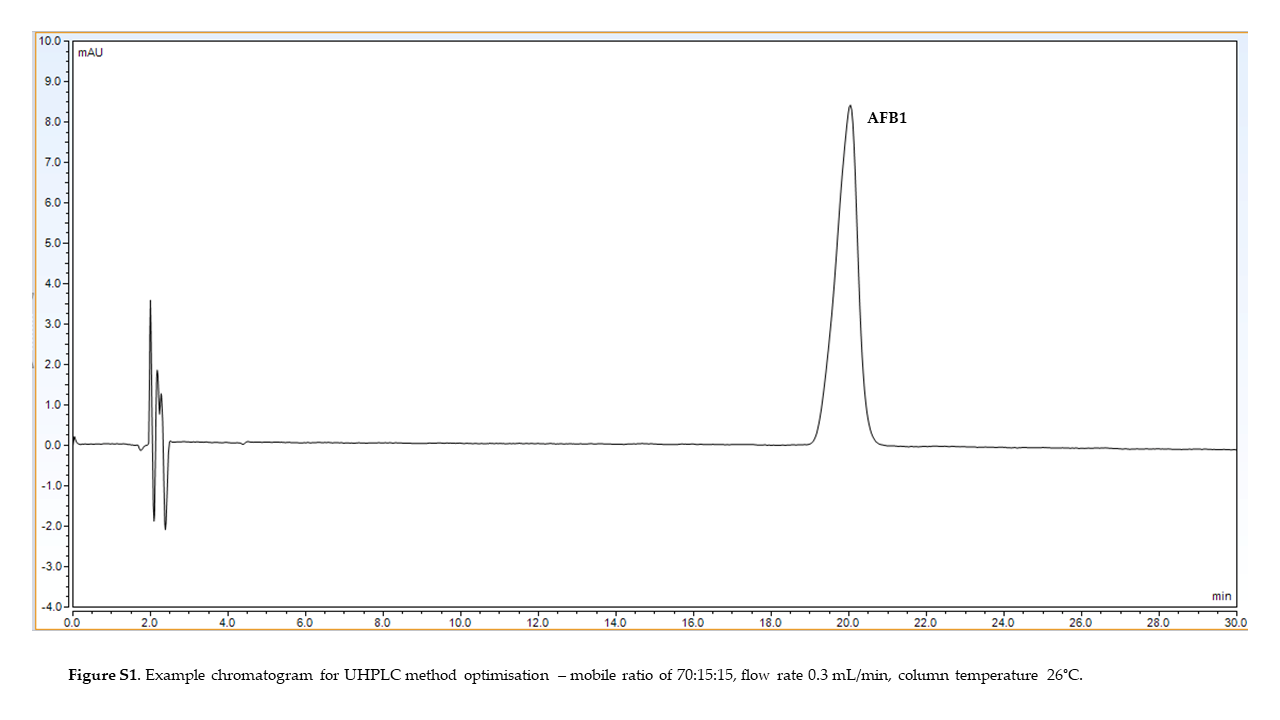

Supplement: Supplementary file 1 [file toxins-13-00002-s001.zip › Figures S1.tif]

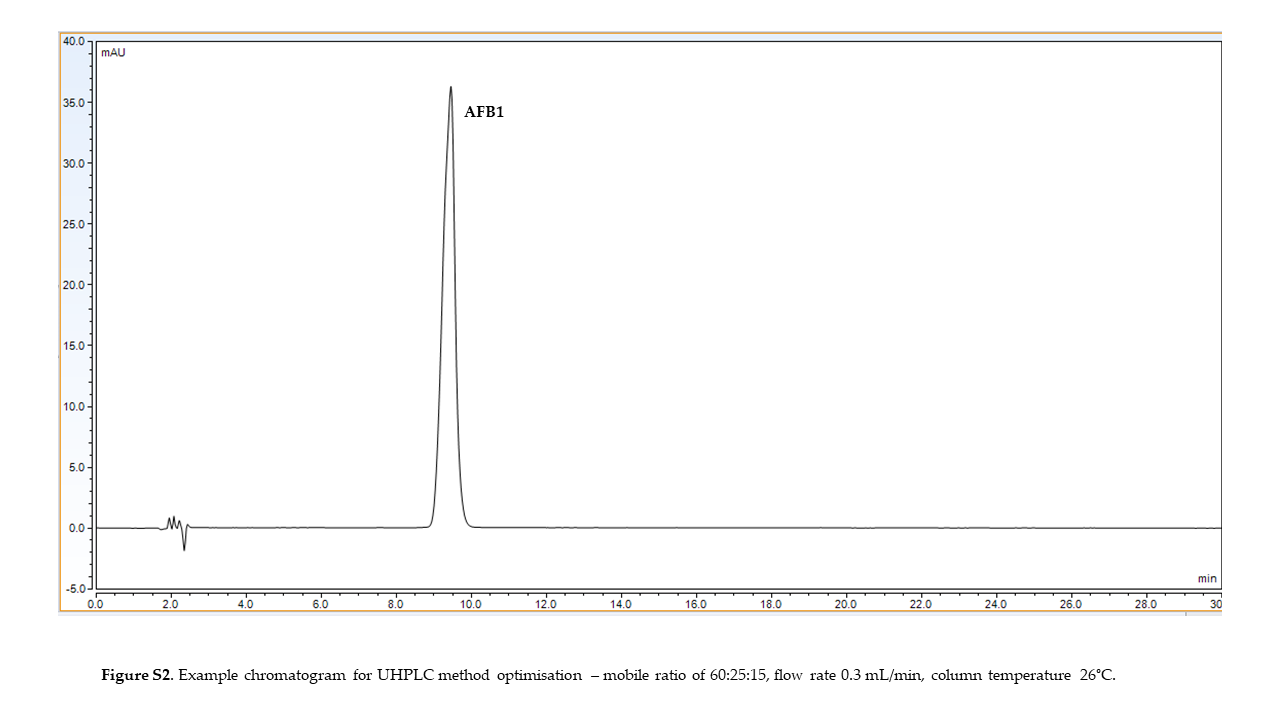

Supplement: Supplementary file 1 [file toxins-13-00002-s001.zip › Figures S2.tif]

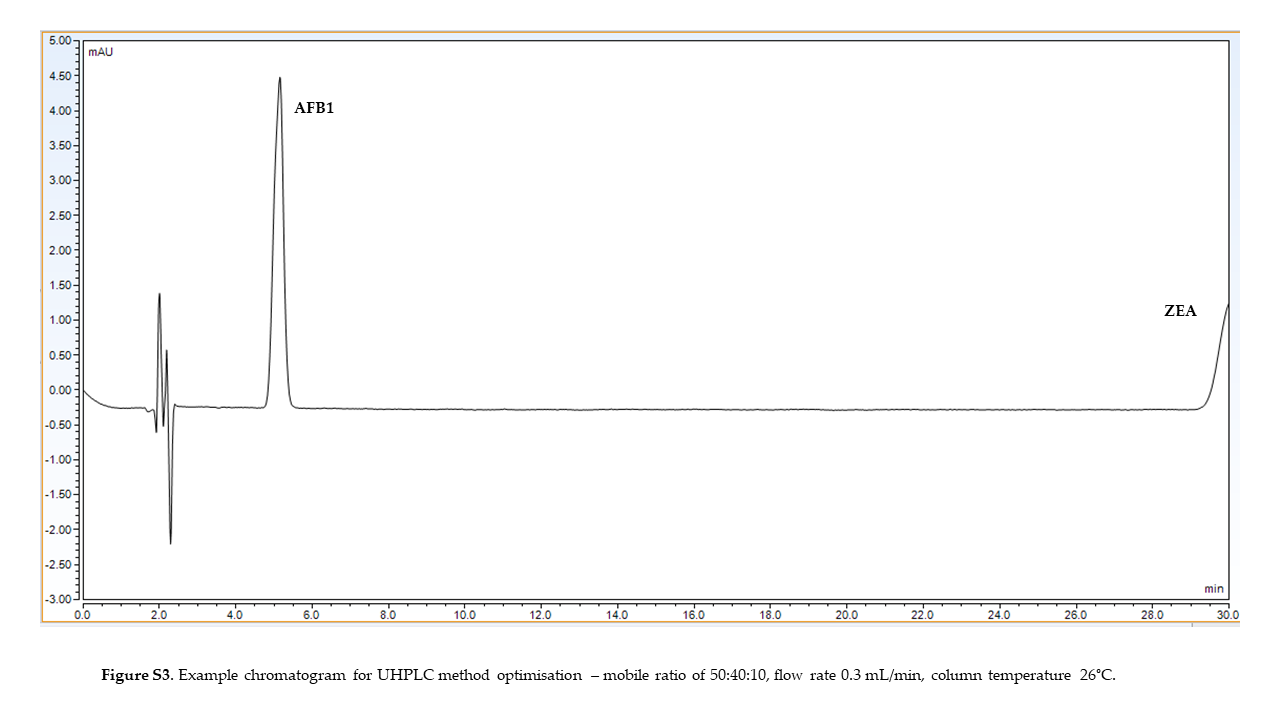

Supplement: Supplementary file 1 [file toxins-13-00002-s001.zip › Figures S3.tif]

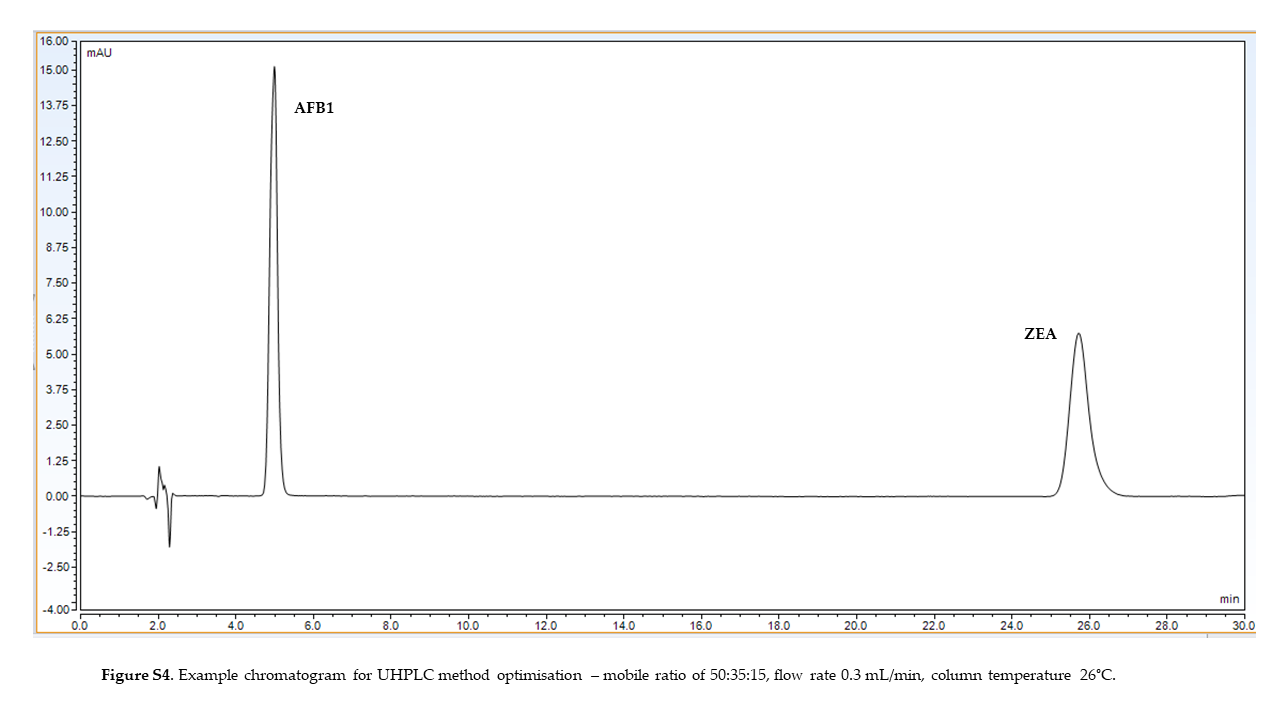

Supplement: Supplementary file 1 [file toxins-13-00002-s001.zip › Figures S4.tif]

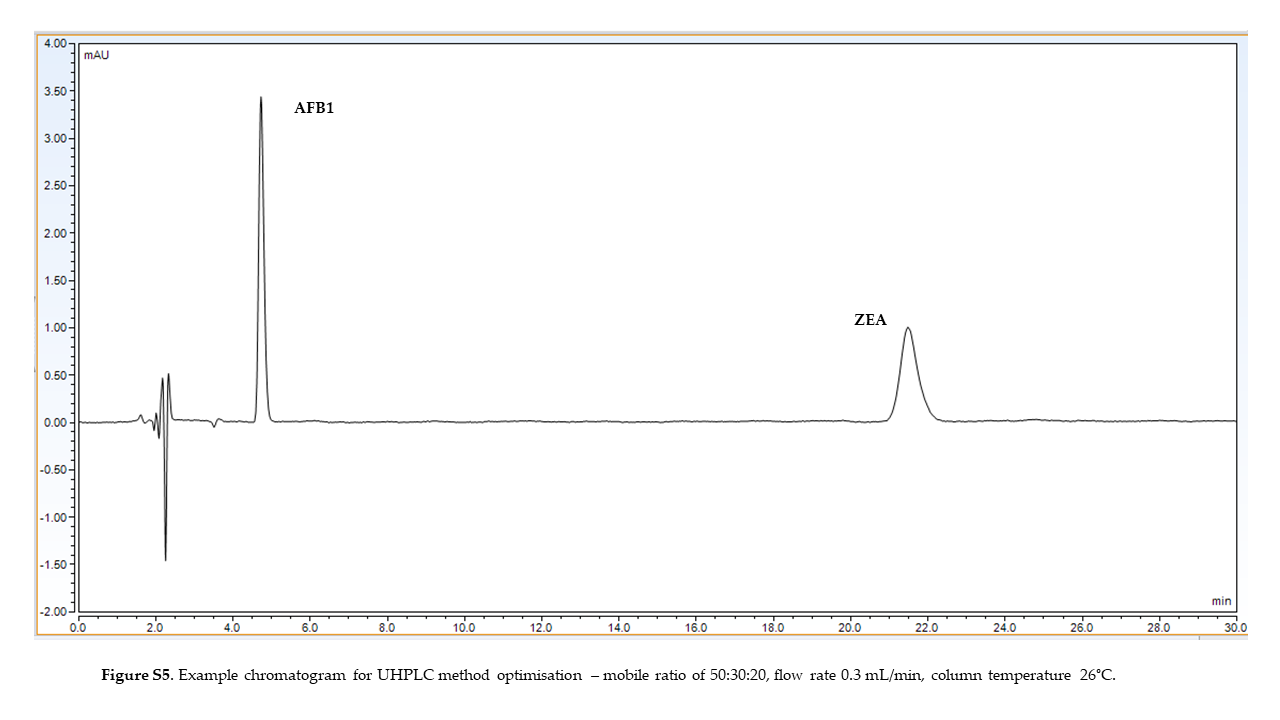

Supplement: Supplementary file 1 [file toxins-13-00002-s001.zip › Figures S5.tif]

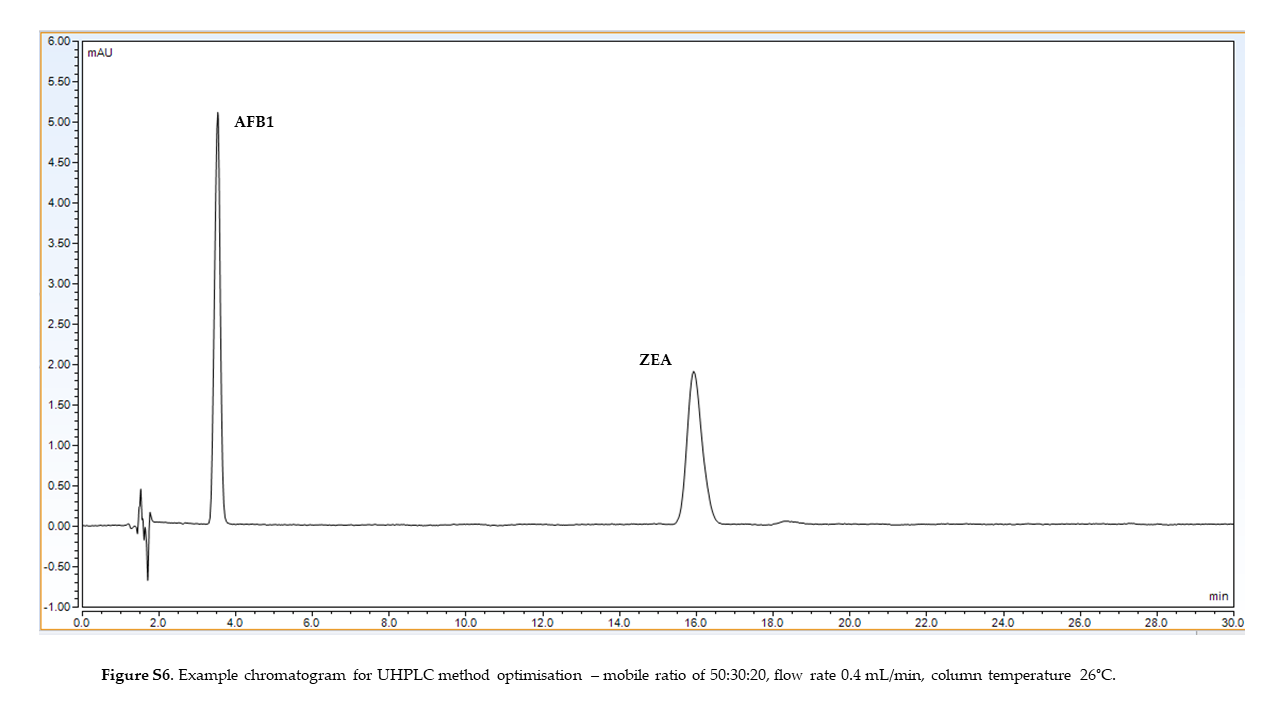

Supplement: Supplementary file 1 [file toxins-13-00002-s001.zip › Figures S6.tif]

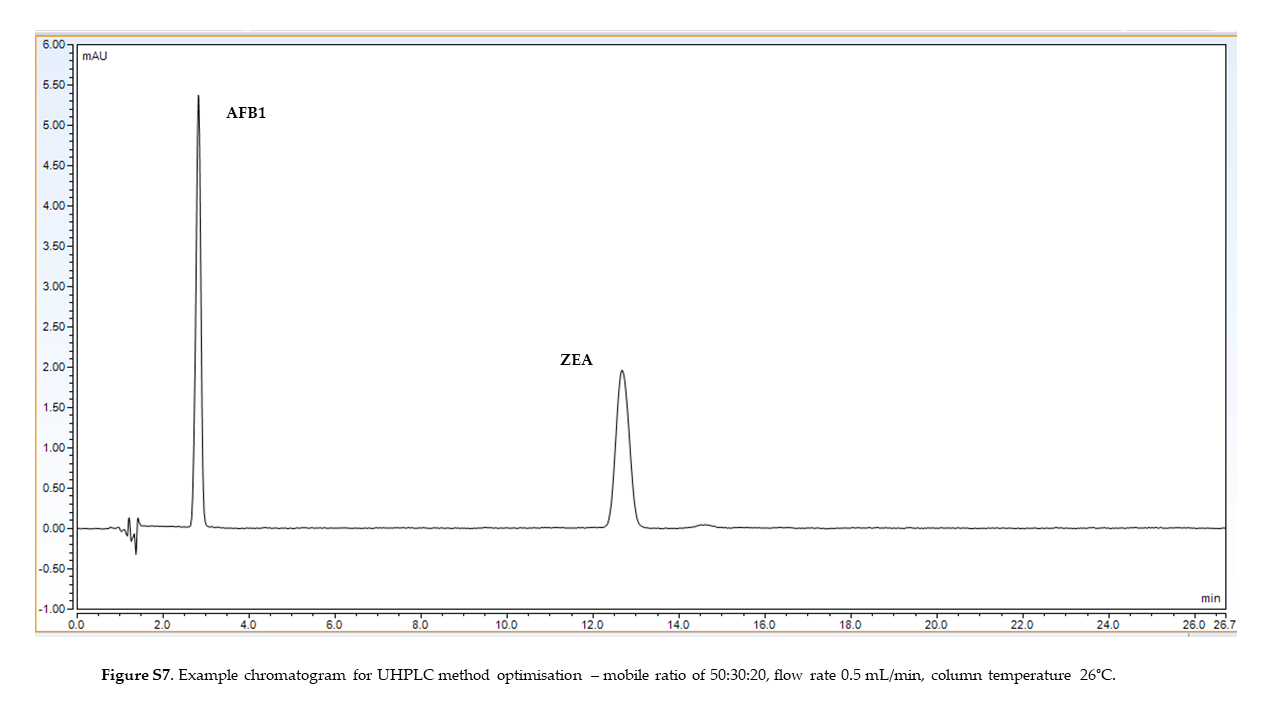

Supplement: Supplementary file 1 [file toxins-13-00002-s001.zip › Figures S7.tif]

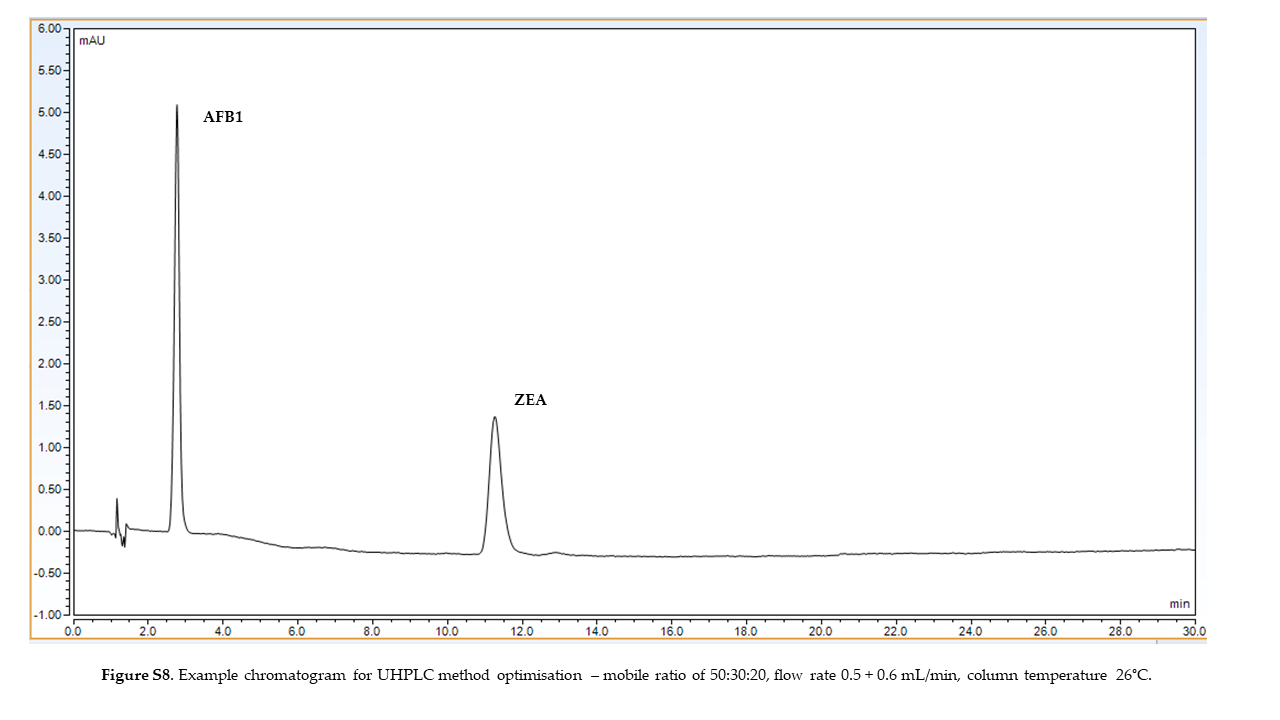

Supplement: Supplementary file 1 [file toxins-13-00002-s001.zip › Figures S8.tif]

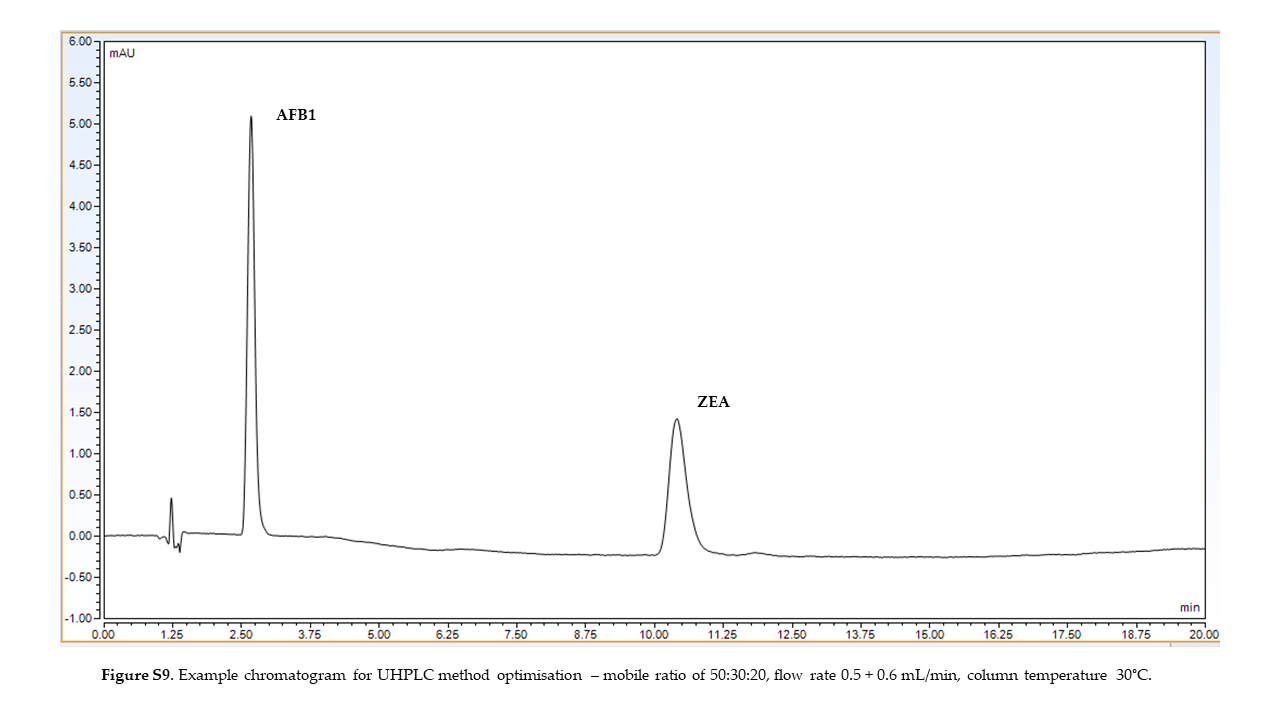

Supplement: Supplementary file 1 [file toxins-13-00002-s001.zip › Figures S9.tif]

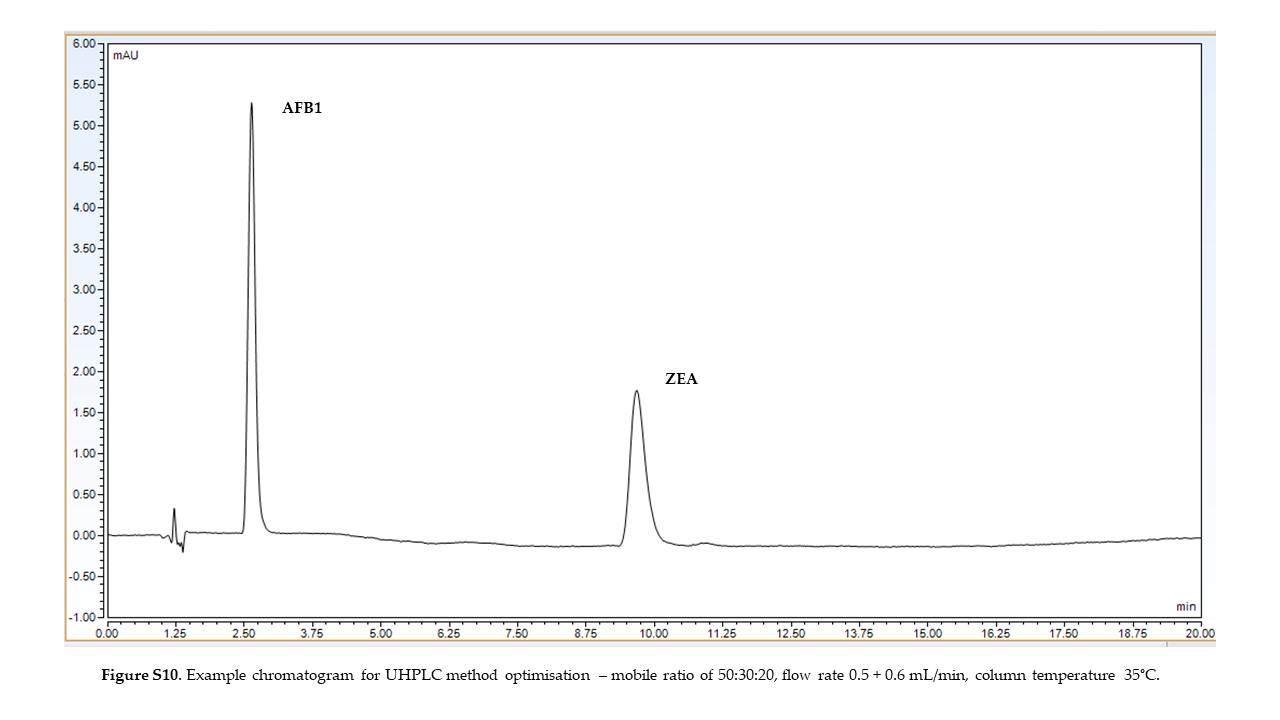

Supplement: Supplementary file 1 [file toxins-13-00002-s001.zip › Figures S10.tif]

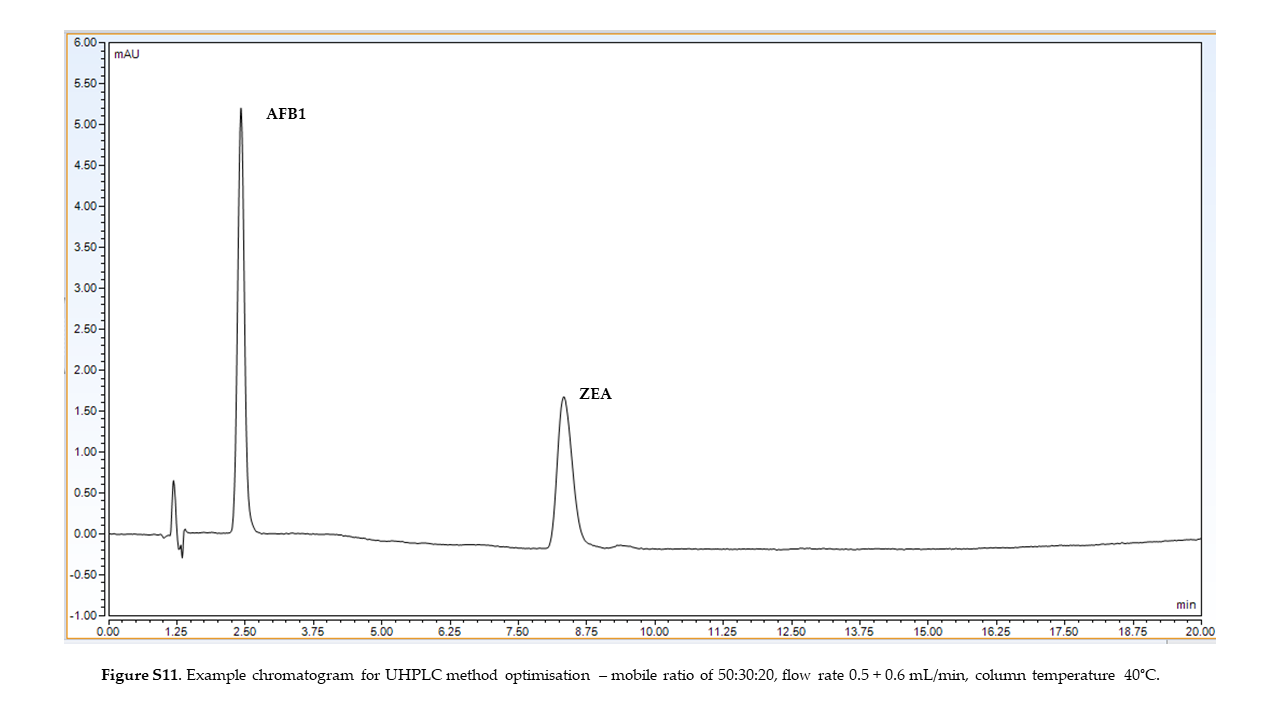

Supplement: Supplementary file 1 [file toxins-13-00002-s001.zip › Figures S11.tif]

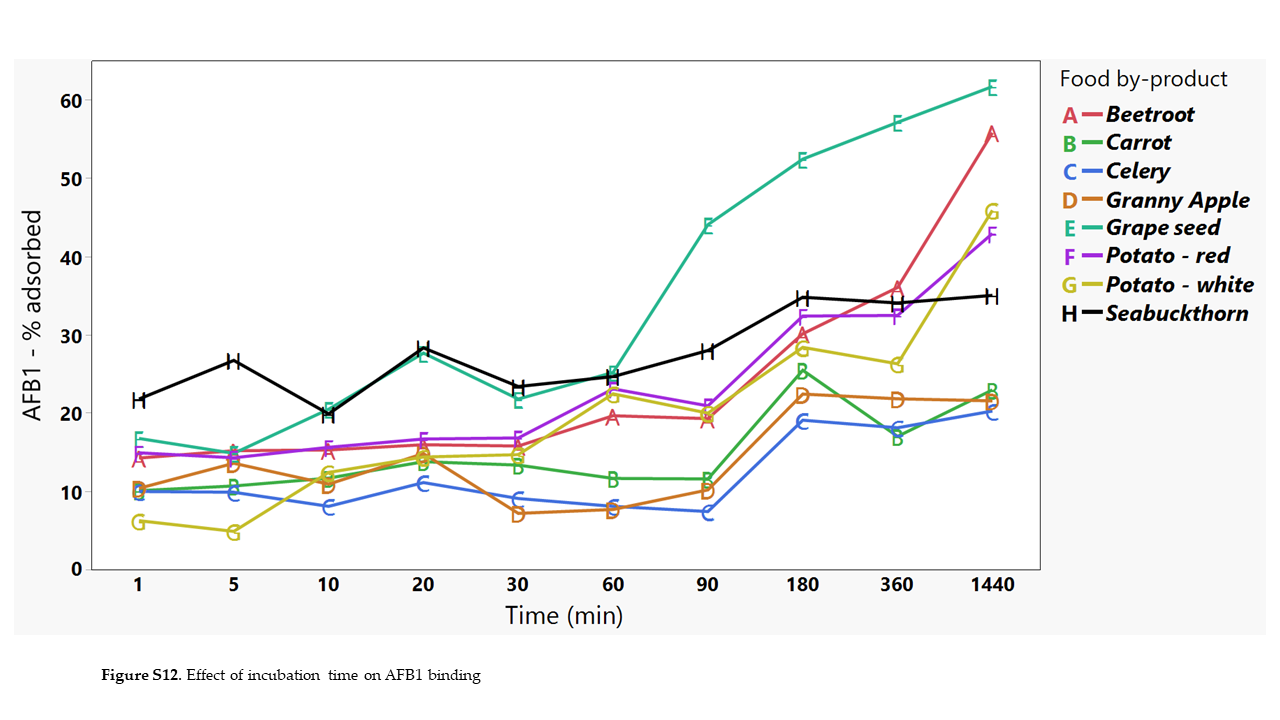

Supplement: Supplementary file 1 [file toxins-13-00002-s001.zip › Figures S12.tif]

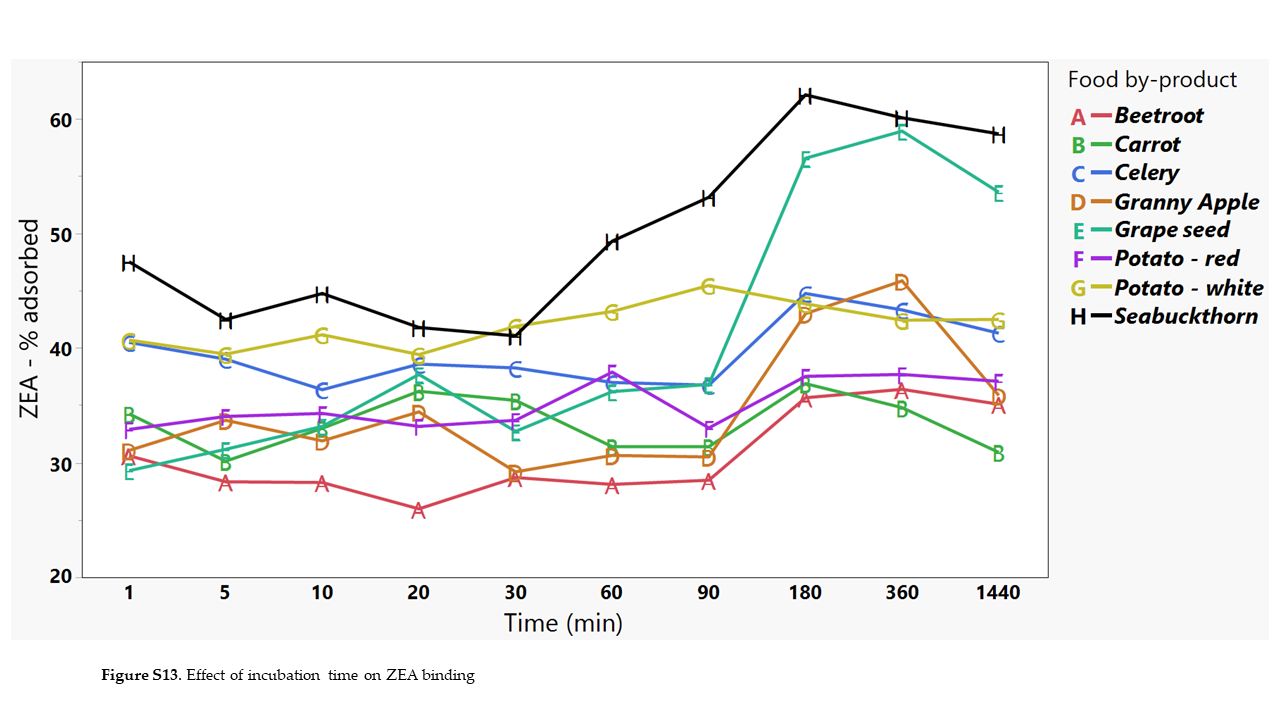

Supplement: Supplementary file 1 [file toxins-13-00002-s001.zip › Figures S13.tif]

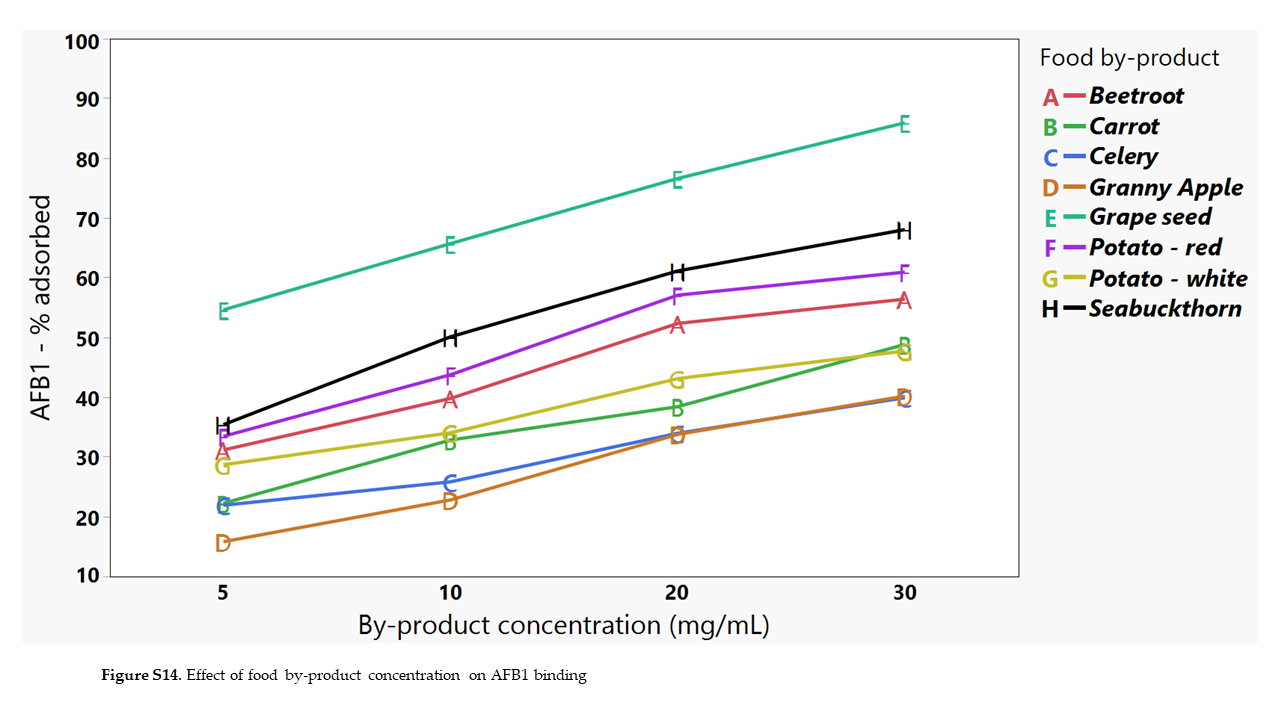

Supplement: Supplementary file 1 [file toxins-13-00002-s001.zip › Figures S14.tif]

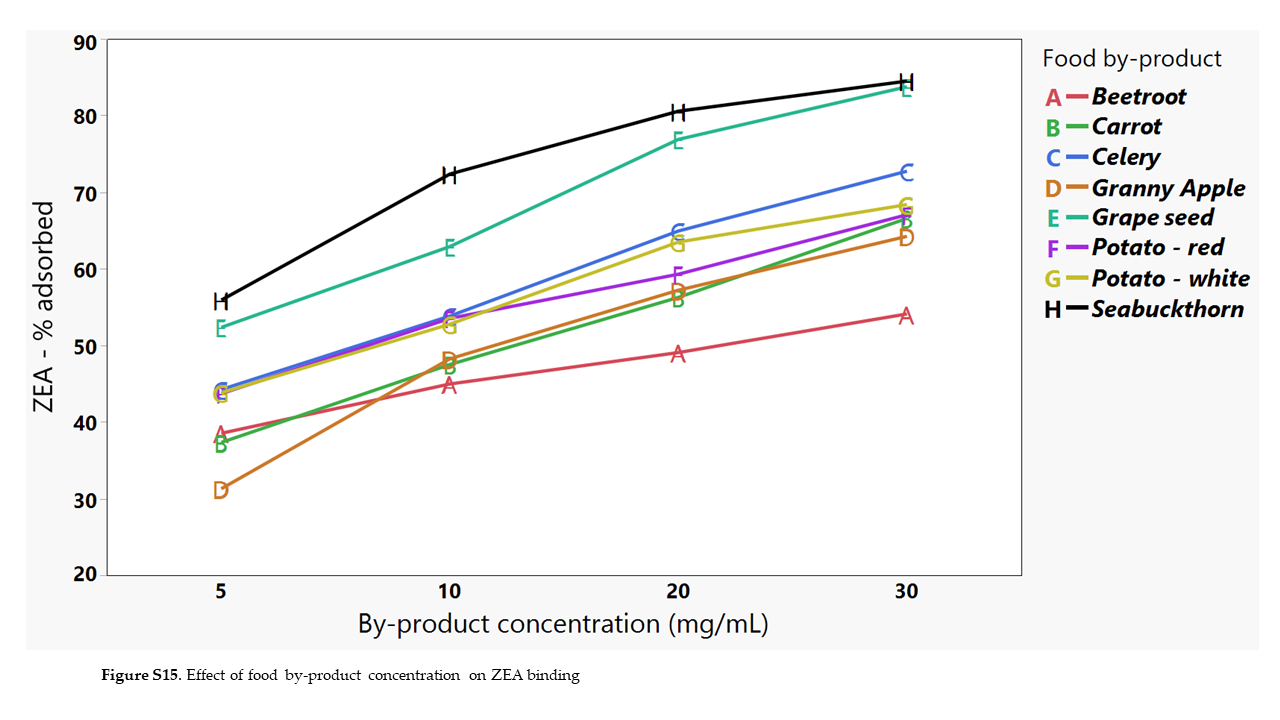

Supplement: Supplementary file 1 [file toxins-13-00002-s001.zip › Figures S15.tif]
